# The genetic repertoire underlying electrogenic sulphur oxidation in cable bacteria

**DOI:** 10.1186/s12864-026-12675-1

**Published:** 2026-03-11

**Authors:** Anwar Hiralal, Jeanine S. Geelhoed, Sinje Neukirchen, Val Karavaeva, Filipa L. Sousa, Filip J. R. Meysman

**Affiliations:** 1 Geobiology Research Group, University of Antwerp, Antwerp, Belgium; 2 Department of Functional and Evolutionary Ecology, University of Vienna, Vienna, Austria

**Keywords:** Cable bacteria, Comparative genomics, Gene expression, Sulphide:quinone oxidoreductase (SQR), Persulphide dioxygenase (PDO), Sulphur disproportionation, YTD-cluster, DsrEFH-like

## Abstract

**Background:**

Cable bacteria are filamentous, sulphur-oxidizing microorganisms of the *Desulfobulbaceae* family that conduct electrons over centimetre-scale distances, coupling sulphide oxidation in deeper sediments to oxygen reduction near the surface. Geochemical evidence demonstrates high rates of aerobic sulphide oxidation in sediments inhabited by cable bacteria. Still, the underlying physiological and molecular basis of this electrogenic sulphur metabolism remains unresolved. Previous genomic analysis proposed that cable bacteria oxidize sulphide by reversing the canonical dissimilatory sulphite reduction (Dsr) pathway.

**Results:**

We evaluated the sulphur metabolism of cable bacteria and related *Desulfobulbales* through comparative genomics, using an expanded set of 31 quality-filtered cable bacteria genomes, including 7 closed assemblies. We showed that cable bacteria encode a complete Dsr pathway, including the previously missing *dsrD* and* dsrT* genes, as well as a novel gene cluster with DsrOP homologues. All Dsr genes were classified as reductive-type, and phylogenetic analyses indicated a close affiliation with those of other *Desulfobulbaceae* (sulphate-reducing and/or sulphur-disproportionating bacteria). In addition, several other previously unrecognized sulphur-metabolism genes were identified in both cable bacteria and closely related *Desulfobulbales*, including a novel subtype of sulphide:quinone oxidoreductase (SQR), a putative rhodanese–persulphide dioxygenase fusion (Rho–PDO), and a YTD gene cluster (consisting of five genes) previously proposed to be characteristic of sulphur-disproportionation lineages. Structural predictions indicate that three uncharacterized YTD-encoded proteins assemble into a DsrEFH-like double heterotrimer, albeit with highly divergent, non-orthologous sequences. Finally, we integrated publicly available transcriptomic and proteomic data to confirm the in vivo expression of these genes, with expression patterns mirroring those of *Desulfolithobacter dissulfuricans* and *Desulfurivibrio alkaliphilus*.

**Conclusion:**

Cable bacteria show minimal genetic divergence and little differential expression in their sulphur-metabolism genes compared to related organisms. Together, our findings challenge the idea that sulphide oxidation occurs via a reversed Dsr pathway. We propose a unique sulphur metabolism model for cable bacteria, in which a canonical reductive/disproportionating sulphur-metabolism repertoire (similar to *Desulfolithobacter dissulfuricans* and *Desulfurivibrio alkaliphilus*) is coupled to net sulphide oxidation through long-distance electron transport. Key steps include sulphide oxidation to polysulphide by SQR, putative conversion to sulphite via Rho–PDO and/or proteins encoded in the YTD cluster, and subsequent disproportionation through the Dsr pathway, where sulphide re-enters the cycle. Net sulphide oxidation and sulphate production arise because electrons are efficiently drained via long-distance electron transport, effectively coupling the metabolism to oxygen reduction.

**Supplementary Information:**

The online version contains supplementary material available at 10.1186/s12864-026-12675-1.

## Introduction

Sulphur oxidation is a key environmental process primarily carried out by microbes. Chemolithotrophic bacteria oxidize reduced sulphur compounds (such as sulphide, elemental sulphur, or thiosulphate) coupled to the reduction of electron acceptors like oxygen or nitrate [1, 2]. Several enzymatic pathways mediate this redox process in sulphur-oxidizing bacteria (SOB), including: (1) the Sox pathway, which uses SoxYZ to bind and oxidize reduced sulphur compounds to sulphate using a complex machinery of proteins [3, 4]; (2) the reverse dissimilatory sulphate reduction (rDsr) pathway, originally described in *Allochromatium vinosum*, which inverts the canonical Dsr pathway of sulphate reducers using specialized sulphur transferases [5–7]; (3) the SQR-mediated pathway, found in heterotrophic sulphide oxidizers, where sulphide:quinone oxidoreductase (SQR), persulphide dioxygenase (PDO), and rhodanese enzymes oxidize sulphide to polysulphides, sulphite, or thiosulphate [8–10]; and (4) the direct oxidation of sulphite to sulphate via molybdenum-dependent sulphite oxidases such as SorAB as found in *Starkeya novella* [11, 12].

In most sediments, oxygen penetrates only a few millimetres below the surface, thus restricting aerobic sulphide oxidation to a narrow oxic–sulphidic interface. This tight redox zonation induces fierce competition and favours the evolution of metabolisms that allow a spatial and/or temporal separation between electron acceptors and reduced sulphur compounds [1]. Some microorganisms have evolved ways of storing the electron acceptor (nitrate) intracellularly, as observed in *Beggiatoa* and *Thiomargarita* species [13, 14]. Cable bacteria, however, have evolved a fundamentally different strategy: long-distance electron transport. Cable bacteria are filamentous organisms and conduct electrons over centimetre-scale distances, coupling sulphide oxidation in deeper anoxic zones to oxygen reduction near the sediment surface [15, 16]. This process, termed electrogenic sulphide oxidation [17], is widely observed in marine and freshwater sediments containing active cable bacteria, where it exerts a strong impact on local biogeochemical cycling [17–21]. The long-distance electron transport itself is mediated by a network of conductive periplasmic fibres that traverse the entire length of the filament [22–25].

Cable bacteria comprise two main genera: *Candidatus (Ca.)* Electrothrix and *Ca.* Electronema [26, 27]. They belong to the *Desulfobulbaceae* family, which also includes the closely related genera *Desulfobulbus*, *Desulfogranum*, and *Desulfolithobacter*, whose members are exclusively single-celled sulphate reducers and sulphur disproportionators [28–30]. At a broader taxonomic scale, cable bacteria fall within the *Desulfobulbales* order, a lineage also largely composed of sulphate-reducing and disproportionating bacteria [31], with two important exceptions: *Desulfurivibrio alkaliphilus* and the CB1MN strain, which can couple sulphide oxidation to nitrate reduction [32, 33]. These exceptions, however, are more distantly related to *Ca.* Electrothrix and *Ca.* Electronema, raising a key question: how did cable bacteria adapt to perform electrogenic sulphide oxidation within such a dominant sulphate-reducing and disproportionating family of organisms?

A pioneering genomic study of cable bacteria was based on six incomplete draft genomes [34] and proposed that cable bacteria oxidize sulphide by reversing the canonical Dsr pathway. The principal argument for this was that cable bacteria lack key genes from the Sox and SorAB systems, but retain the central Dsr machinery (*dsrAB*, *dsrMK*, *sat*, *aprAB*, *qmoABC*). The presence of these genes was confirmed in subsequent studies with closed genomes of *Ca*. Electronema [35], but several Dsr-associated genes (*dsrJOP*, *dsrD*, *dsrT*) were either reported missing or not annotated. In contrast, it was recently shown that the complete genome of *Ca*. Electrothrix scaldis GW3-3 contains several additional *dsr*-related genes [36], including *dsrD*, a putative functional marker and allosteric activator of DsrAB sulphite reductase activity [37, 38].

Here, we analyse an expanded genomic dataset comprising all currently available cable bacteria genomes [35, 36, 39–43]. We systematically identify sulphur-metabolism genes, including several previously unreported candidates, and use phylogenetic analyses to place them in an evolutionary context. Furthermore, we integrate transcriptomic and proteomic data from cable bacteria and their close relatives to evaluate the in vivo expression of these genes. Together, our results provide a revised molecular model for sulphide oxidation in cable bacteria.

## Materials and methods

### Cable bacteria genome dataset

Previously published cable bacteria genomes were identified [34–36, 39–43] and their GenBank files were downloaded from the National Centre for Biotechnology Information (NCBI) database, resulting in a total set of 53 genomes. In addition, nineteen genomes of related species within the *Desulfobulbales* order were downloaded from the NCBI database (accessed on the 5th of January 2024) as references. GenBank annotations were used for all genomes except those for which annotations were unavailable (see Table S1). In the latter case, Prodigal v2.6.3 and Prokka v1.14.5 were used with standard parameters for gene identification and annotation, respectively [44, 45]. CheckM2 v1.02 was used to assess genome completeness and contamination. Genomes with completeness below 75% or contamination above 5% were not used for further analysis (thus reducing the cable bacteria set to 31 genomes). See Table S1 for an overview of the used genome dataset.

Eighteen cable bacteria genomes were selected as species representatives based on completeness and contamination criteria (Table S1). Amino acid identity calculations were performed using CompareM v0.1.2 (https://github.com/donovan-h-parks/CompareM). Phylogenomic analysis was performed using GTDB-Tk2 v2.4.1 [46], which identified and aligned 120 bacterial marker genes across the cable bacteria representatives, the related *Desulfobulbales* (Table S1), and *Geobacter sulfurreducens* PCA (used as an outgroup; Table S1). A maximum-likelihood phylogeny was inferred with IQTREE v2.1.4 [47] using ModelFinder for model selection [48] and 100 standard non-parametric bootstrap replicates. The resulting phylogenetic reconstruction was visualized with FigTree v1.4.4 (http://tree.bio.ed.ac.uk/software/figtree/).

### Identification of sulphur metabolism genes

All genomes in the dataset were screened for sulphur-metabolism genes using a combination of the DiSCo [49] and HMSS2 [50] search tools. Filtered output of both programs was used to identify most of the genes belonging to the Dsr pathway (except for *dsrOP* homologues, which were not identified in the filtered output of these tools). In addition, DiSCo was used to categorize these genes as reductive or oxidative type [49]. Since DsrOP homologues were previously identified in the genome of *Ca*. Electrothrix scaldis GW3-3 [36], we used blastp v.2.12.0 with filtering for an E-value < 10^− 15^ and a minimum alignment length of 70% to detect these genes in the current genome dataset.

Other proteins related to sulphur metabolism were identified in the raw output of HMSS2, including the sulphate permease SulP, periplasmic and cytoplasmic rhodaneses, the thiosulphate/polysulphide reductase complex Phs/PsrABC, sulphide:quinone oxidoreductase (SQR), the putative rhodanese-persulphide dioxygenase (Rho-PDO) fusion protein and two genes of the YTD cluster (*tusA*,* dsrE-like*) [51]. Additional genes were identified through synteny (gene neighbourhood) analysis, which included other YTD cluster genes (*yeeE*, *chp1*, *chp2*) and a small membrane protein and a tetrahaem-binding-site-containing-gene near *dsrOP* homologues.

Identification of protein sequence searches was complemented by determining putative protein domains using the web-based version of InterProScan (accessed throughout March-June 2024) and comparing these to characterised proteins [52]. Signal peptides were predicted using SignalP v6.0 [53], and transmembrane helices using DeepTMHMM v1.0 [54]. Multimer predictions were performed using AlphaFold2-Multimer to infer the oligomeric states of the DsrEFH-like protein complex and the TMH_1_–DsrOhPh–tetrahaem proteins [55]. In addition, structural models for all other proteins were generated using AlphaFold3 [56]. Where applicable, relevant cofactors and ions were included. Default parameters were used, and the highest-confidence model was selected. The predicted multimer-protein structure of the DsrEFH-like protein was compared against the *Allochromatium vinosum* DsrEFH crystal structure (PDB: 2HY5) using the DALI server [57].

The proposed electrogenic sulphur metabolism model for cable bacteria was constructed by integrating the comparative genomic and gene expression analyses (see below) in this study. Genes with putative roles in sulphur metabolism were included only if they were conserved across the majority of cable bacteria lineages and detected as expressed. Corresponding reaction schemes were assigned based on KEGG pathway annotations and supported by curated evidence from the literature.

### Phylogenetic analysis

For the protein sequences of DsrABCDEFHTMKJOP, QmoABC and AprAB, we used previously verified sequences as reference sequences to build the alignment [58]. In addition, other sulphur metabolism and *dsrOP* homologues were used as queries for blastp searches against the RefSeq database (accessed throughout March-June 2024), and hits were filtered for a minimum E-value of 10^−10^ and minimum alignment length of 70% for a maximum of 500 sequences, with the hits used to build the alignment. An extra dataset was created for the DsrP homologue using a previously described collection of NrfD family protein sequences [59], which were used as reference sequences to build the alignment.

Sequences with multi-pass transmembrane helices were aligned using the structural alignment tool Praline [60] with standard parameters, as its profile-based strategy incorporates predicted structural features to improve alignment of membrane proteins. All other sequences were aligned using Clustal Omega v.1.2.3 [61] with the following parameters: --max-guidetree-iterations=100, --max-hmm-iterations=100, --output-order=tree-order. Alignments were trimmed using trimAl v1.4.1 [62] with a gap threshold of 0.95 and 60% of the positions in the original alignment to be conserved. IQTree v2.1.4 [47] was used to calculate all maximum-likelihood phylogenies using the ModelFinder option [48] and 1000 UltraFast Bootstraps using UFBoot2 [63]. Phylogenies were visualized using FigTree v1.4.4 (http://tree.bio.ed.ac.uk/software/figtree/).

### Gene expression analysis using published transcriptomics and proteomics data

Two metatranscriptome datasets for *Ca.* En. aureum were obtained from the Short Read Archive. Metatranscriptome analysis was performed on sediment-based enrichment cultures with freshwater sediment. These enrichment incubations were performed at 15 °C, either under oxic conditions (PRJNA575166) or under nitrate-reducing conditions (PRJNA575156), as described previously in detail in [64]. The reads were mapped to the coding genes of *Ca*. En. aureum (GCA_942492785.1) using the seal tool implemented in bbmap v39.06 (https://sourceforge.net/projects/bbmap/ https://jgi.doe.g.ov/data-and-tools/software-tools/bbtools/bb-tools-user-guide/seal-guide/) with default settings. Gene expression was calculated as TPM = (reads mapped per gene/gene length)/Sum(reads mapped per gene/gene length) for each replicate (*n* = 3) and averaged per treatment. 

A metatranscriptome dataset for *Desulfurivibrio alkaliphilus* was downloaded from the short read archive (PRJNA322753). Axenic cultures of were grown anaerobically at 30 °C in an alkaline, carbonate-buffered mineral medium (pH 9.5) under sulphide-oxidizing conditions with nitrate or under sulfur-disproportionating conditions, as described previously in detail in [33]. Reads were mapped to the genome of *Desulfurivibrio alkaliphilus* (GCF_000092205.1) and TPM values were calculated for the three replicates per growth condition and averaged.

Proteomic datasets for *Desulfolithobacter dissulfuricans* were retrieved from a previous study [30]. An axenic culture was grown at 35 °C under thiosulphate-disproportionating or sulphate-reducing conditions [30]. Protein-coding sequences of interest were identified in the genome (GCA_025998535.1), and the corresponding protein abundances retrieved from literature. Protein abundance was calculated as NSAF = (number of spectral counts per protein/protein length)/Sum(spectral counts per protein/protein length) for each of the three replicates per growth condition and averaged.

## Results and discussion

### Cable bacteria possess a reductive-type Dsr pathway similar to other members of the *Desulfobulbaceae* family

A total of 53 publicly available cable bacteria genomes were quality-checked and filtered for completeness (> 75%) and contamination (< 5%), yielding 31 quality-filtered genomes representing 18 species (Table S1). Among these, seven genomes were closed, enabling the most stringent verification of gene presence or absence.

**Fig. 1:**
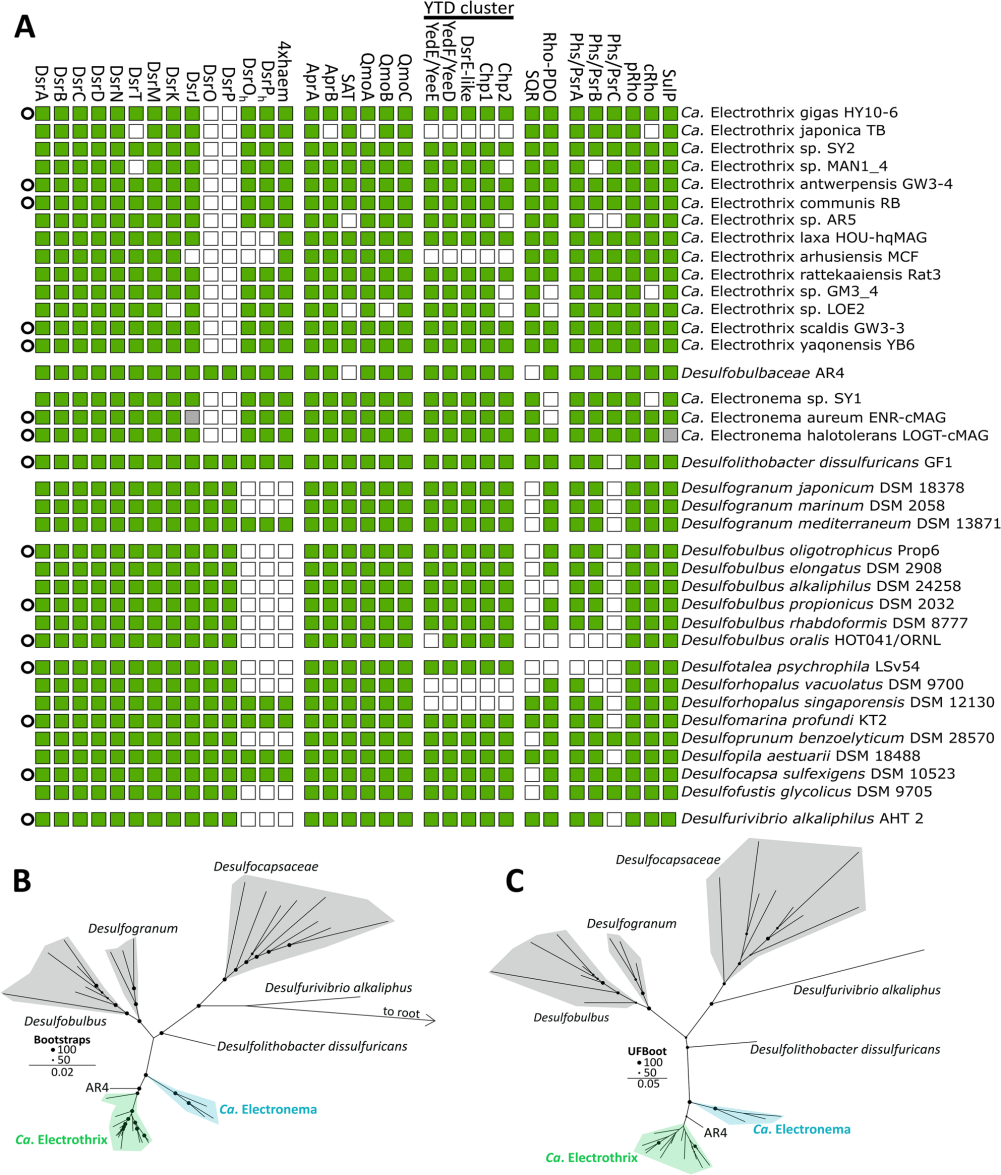
Sulphur metabolism genes in cable bacteria and related members of the *Desulfobulbales* order. **A** Presence/absence of sulphur metabolism-related genes identified in this study. Presence is indicated with filled squares. Note that in some genomes, genes are not properly annotated but identified in the genome sequence using tblastn (grey squares). Closed genomes are indicated with a circle. Only species representatives are shown (for the other genomes, see Table S2). **B** Maximum-likelihood phylogeny (model LG + F+R5) using concatenated GTDB-Tk2 identified marker genes of cable bacteria species representatives and related species within the *Desulfobulbales* order. *Geobacter sulfurreducens* PCA is used to root (not visible). **C** Unrooted maximum-likelihood phylogeny (model LG + F+R4) of concatenated Dsr-pathway genes found in cable bacteria species representatives and related species within the *Desulfobulbales* order

Comparative genomic analysis showed that all closed genomes encode the complete set of core components of the dissimilatory sulphate reduction (Dsr) pathway, including DsrABC, DsrD, DsrN, DsrTMKJ, QmoABC, AprAB, SAT, and sequence-based homologues of DsrOP (Fig. 1A; Table S2). The apparent absence of some of these genes in non-closed genomes is most likely attributable to genome incompleteness.

Notably, *dsrD* and *dsrT* genes (two genes previously unreported in cable bacteria) were detected in all closed and high-quality genomes (Fig. 1A). DsrT is typically found in organisms with reductive-type Dsr enzymes [58], and is thought to play a regulatory role in the Dsr pathway gene expression [65]. DsrD was recently shown to act as an allosteric activator of DsrAB sulphite reductase activity [37, 38], and is considered an indicator of reductive sulphur or disproportionating metabolism, when co-occurring with *dsrC*, *dsrMK*, and *dsrAB* genes [37]. In contrast, *dsrL*, coding for a mediator of sulphide oxidation in *A. vinosum* [66], was not found in cable bacteria. Although several pyridine nucleotide:disulphide oxidoreductases (the DsrL protein family) are present in cable bacteria, none met the domain architecture requirements (N-terminal ferredoxin, NAD(P)H-binding domain and C-terminal ferredoxin) to be classified as DsrL [67].

To investigate whether cable bacteria have acquired genetic adaptations within their core Dsr pathway, we compared a species-level phylogenomic reconstruction based on concatenated conserved marker genes (identified using GTDB-Tk2; Fig. 1B) with a concatenated Dsr-pathway phylogeny (Fig. 1C) constructed from DsrABCDMKJ, AprAB, SAT, and QmoABC sequences. The resulting phylogenomic topology largely agrees with previous studies [35, 41, 42]. The cable bacteria genera of *Ca*. Electrothrix and *Ca*. Electronema are most closely related to the *Desulfolithobacter* genus based on phylogenomic distance and amino acid identity (Fig. 1B, S1), followed by *Desulfogranum* and *Desulfobulbus* (Fig. 1B). The concatenated Dsr-pathway phylogeny mirrors the species-level relationship, suggesting that the Dsr pathway was most likely vertically acquired from a common ancestor (Fig. 1C).

To further test for functional divergence, we classified individual Dsr proteins as either reductive or oxidative type using HMM-based approaches (DiSCo and HMSS2) [49, 50]. All Dsr proteins identified in cable bacteria were classified as reductive type, a result confirmed by phylogenetic analyses of each Dsr component using extended reference datasets [58]. In every case (including DsrAB, DsrC, DsrTMKJ, DsrD, AprAB, and QmoA) cable bacteria sequences clustered within the reductive clades, typically alongside other *Desulfobulbaceae* members (Fig. S2–S14). The only exceptions were the *DsrOP* homologues, which formed a separate but non-oxidative type cluster (Figure S13-14). DsrAB sequences from *Desulfurivibrio (Dv.) alkaliphilus*, an distantly related organism capable of sulphide oxidation via nitrate reduction [33], remain phylogenetically distinct from those of the *Desulfobulbaceae* family (Fig. S16-S17). Hence, the phylogenetic placement of individual Dsr components also mirrors the species-level phylogenomic relationship of cable bacteria and their close relatives, indicating vertical inheritance of the Dsr pathway in cable bacteria, with minimal genetic adaptation.

### Identification of a distinct locus with *dsrOP* homologues and a tetrahaem-containing protein

**Fig. 2:**
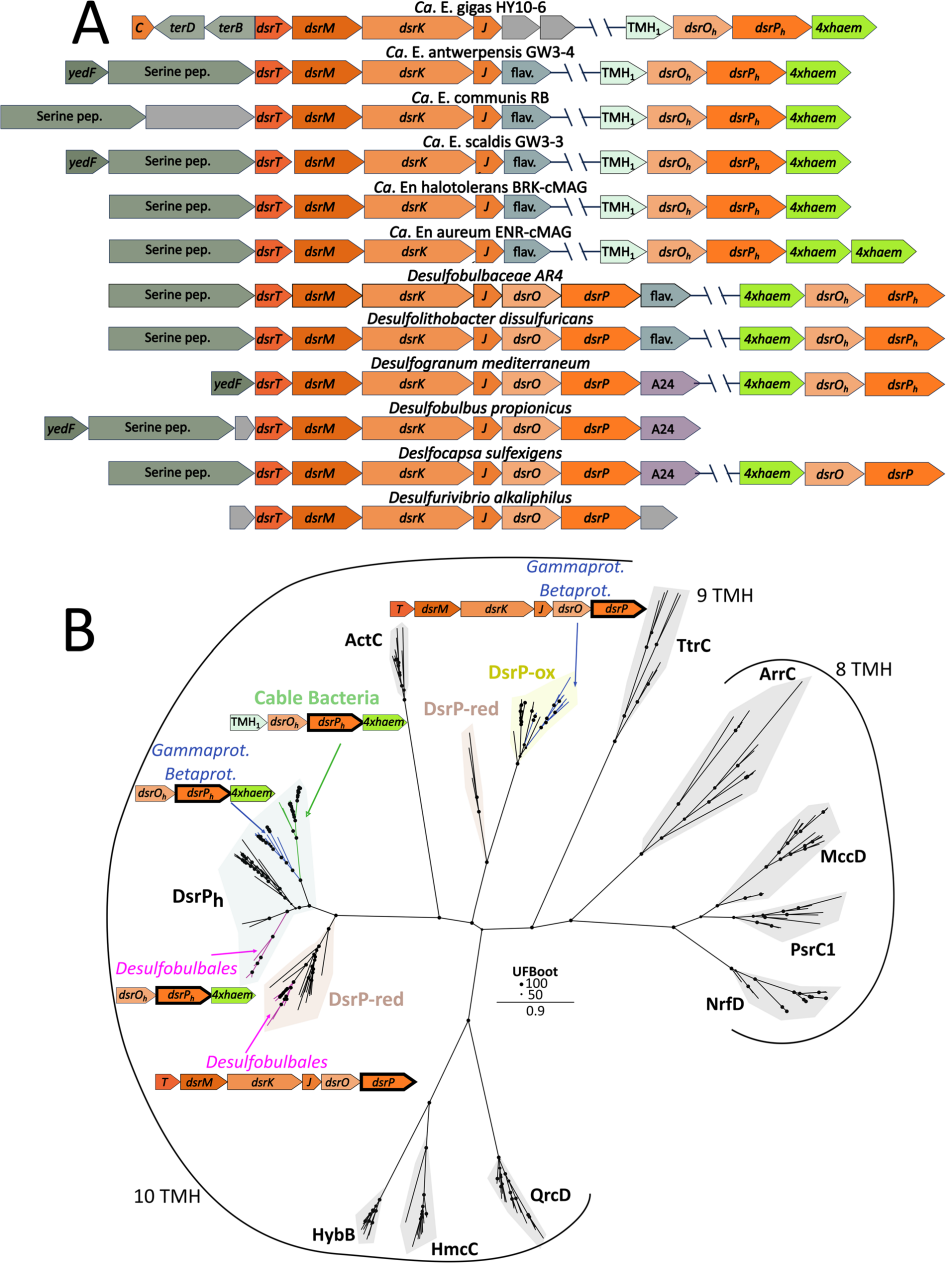
Putative DsrOP homologues in cable bacteria are located on a separate *dsrO*_*h*_*P*_*h*_*-tetrahaem* locus. **A** Operon structure of the *dsrTMKJ(OP)* locus in cable bacteria and species representatives of *Desulfobulbales* genera. Note that not all species within the *Desulfobulbales* order possess the *dsrO*_*h*_*P*_*h*_*-tetrahaem* cluster (Table S2). **B** Unrooted maximum-likelihood phylogeny (model LG + F+I+G4) of DsrP_h_ proteins, canonical DsrP sequences, RefSeq hits and NrfD-family proteins. Non-DsrP NrfD family clusters are indicated in grey. Cable bacteria DsrP_h_ sequences cluster basally to a reductive-type canonical DsrP branch. Several *Desulfobulbales* (pink; excluding cable bacteria) and Refseq hits of the *Gamma*-and *Betaproteobacteria* classes (blue) have genomes containing putative canonical DsrP (in the *dsrTMKJOP* locus) and DsrP_h_ (in the *dsrO*_*h*_*P*_*h*_*-tetrahaem* locus)

In most sulphate-reducing bacteria, *dsrTMKJOP* genes are co-located within a single operon encoding DsrT and the DsrMKJOP complex, which transfers electrons from the quinone pool to cytoplasmic DsrC-trisulphide [68]. In *Ca*. Electronema and *Ca*. Electrothrix, however, only *dsrTMKJ* are found together in one locus, while homologues of *dsrOP* (*dsrO*_*h*_*P*_h_) are distally located in the genome. The homologues are found together with a gene encoding a predicted periplasmic cytochrome *c* (Fig. 2A), with *Ca*. Electronema being the sole species possessing two copies of this gene (Table S2). Functional annotation and structural prediction of this protein indicate the putative presence of four haem-binding sites (hereafter referred to as tetrahaem), which are all His-His coordinated (Fig. S18). The locus also consistently contains a small gene encoding for a single transmembrane-containing protein (TMH_1_) with no annotated function (Fig. 2A), which is unique to cable bacteria. The genomic organization of two separate loci in cable bacteria contrasts with that of other *Desulfobulbales*, which generally possess a canonical *dsrTMKJOP* operon, though several, including *Desulfolithobacter (Dl.) dissulfuricans* (the closest described relative of cable bacteria) and the hitherto uncharacterized AR3 and AR4 cable bacteria strains, also contain the *dsrO*_*h*_*P*_h_*–tetrahaem* locus (Fig. 2A; Table S2), although no gene encoding the unknown TMH_1_. Importantly, this same TMH_1_-DsrO_h_P_h_-tetrahaem complex has also previously been identified in the genomes of *Ca*. Electrothrix communis and *Ca*. Electronema aureum, where it was speculated to represent a “candidate alternative complex III” [69].

Phylogenetic analyses placed DsrM, DsrK, and DsrJ of cable bacteria firmly within the reductive-type Dsr clades [49] clustering with *Desulfobulbaceae* sequences, although DsrJ showed high sequence divergence (Figure S10-12). In contrast, the DsrO_h_P_h_ and tetrahaem proteins form a separate reductive-type subclade distinct from the *Desulfobulbales* sequences and are more closely related to sequences from *Gammaproteobacteria* and *Betaproteobacteria* (Fig. S13-S15), many of which are known sulphur-oxidisers [70–72] (Table S3). Furthermore, the majority of these organisms contain a full rDsr-pathway (including a canonical *dsrMKJOP* locus) of the oxidative type, as determined using DiSco, in addition to separate *dsrO*_*h*_*P*_h_*-tetrahaem* genes (Table S3). However, 5 out of 16 investigated genomes lack any sulphur metabolism potential (Table S3), but do contain separate *dsrO*_*h*_*P*_h_*-tetrahaem* genes, thus suggesting that this locus in cable bacteria might be involved in a different biological process independent from the DsrMKJOP system.

DsrP belongs to the NrfD family of quinone-binding transmembrane proteins that assemble into modular, energy-transducing complexes with cytochrome-*c*, Fe–S, or molybdopterin-containing partners involved in sulphur, nitrogen, and hydrogen redox processes [73–76]. Based on its predicted modular architecture and predicted structure (Fig. S19), the putative TMH_1_-DsrO_h_P_h_-tetrahaem complex most closely resembles the MccACD sulphite reductase system, or it could be a candidate for alternative complex III as previously suggested [69]. However, phylogenetic analysis of NrfD-family proteins shows that DsrP_h_ sequences from cable bacteria, *Desulfobulbales*, *Gamma*-and *Betaproteobacteria*, cluster near canonical reductive-type DsrP proteins (Fig. 2B), and not with MccD or ActC, which represent the NrfD-family proteins of sulphite reductase and alternative complex III, respectively [59, 76]. In addition, DsrP_h_ sequences form a distinct clade separate from canonical DsrP, while the bona fide DsrP sequences (found in the *dsrMKJOP* loci) of *Desulfobulbales* and *Gamma*-and *Betaproteobacteria* cluster with canonical DsrP sequences of reducers and oxidizers, respectively (Fig. 2B).

Together, based on the distal loci (Fig. 2A), distinct phylogenetic clusters (Fig. 2B) and the presence of the *dsrO*_*h*_*P*_*h*_*-tetrahaem* locus in organisms otherwise lacking canonical Dsr-genes (Table S3), the DsrO_h_P_h_-tetrahaem proteins found in cable bacteria might not be involved in DsrMKJOP complex formation (Fig. S19), but may mediate another metabolic process. Based on its predicted domains and modelled complex (Fig. S19), we speculate that the putative TMH_1_-DsrO_h_P_h_-tetrahaem complex in cable bacteria may be involved in electron transfer from the quinone pool to the periplasmic space, and could be a candidate for transferring electrons to periplasmic cytochromes, a required step for the electron transfer to the conductive fibres [34, 77]. This could be accompanied with proton translocation, as previously suggested for DsrOP proteins [68]. However, the presence of this locus in non-cable bacteria (some of which do not metabolise sulphur at all; Table S3) might indicate an unknown metabolic function. Further biochemical characterisation will be necessary to clarify the role and energetic implications of the putative DsrO_h_P_h_-tetrahaem proteins in cable bacteria.

### Cable bacteria and related *Desulfobulbales* possess a conserved YTD cluster encoding a divergent DsrEFH-like complex

Cable bacteria and related members of the *Desulfobulbales* possess a conserved “YTD cluster” (Fig. 1), previously proposed as a genetic marker for sulphur-disproportionating organisms [51, 78]. The cluster encodes a YedE-related sulphur uptake protein (YeeE), a TusA-like sulphur transferase (YeeD), a DsrE-like protein, and two conserved hypothetical proteins (Chp1 and Chp2) of unknown function [51]. Orthologues of the YedE-related and TusA-family proteins from *Spirochaeta thermophila* have recently been biochemically characterised [79, 80]. YeeE functions as a thiosulphate uptake protein embedded in the cytoplasmic membrane, while YeeD acts in concert with YeeE to bind thiosulphate on a conserved cysteine residue, after which sulphite and sulphide are released through an as-yet-unresolved “decomposition” process. Importantly, YeeD dissociates from YeeE after thiosulphate binding, suggesting that YeeD may also act independently on cytoplasmic thiosulphate [80].

**Fig. 3:**
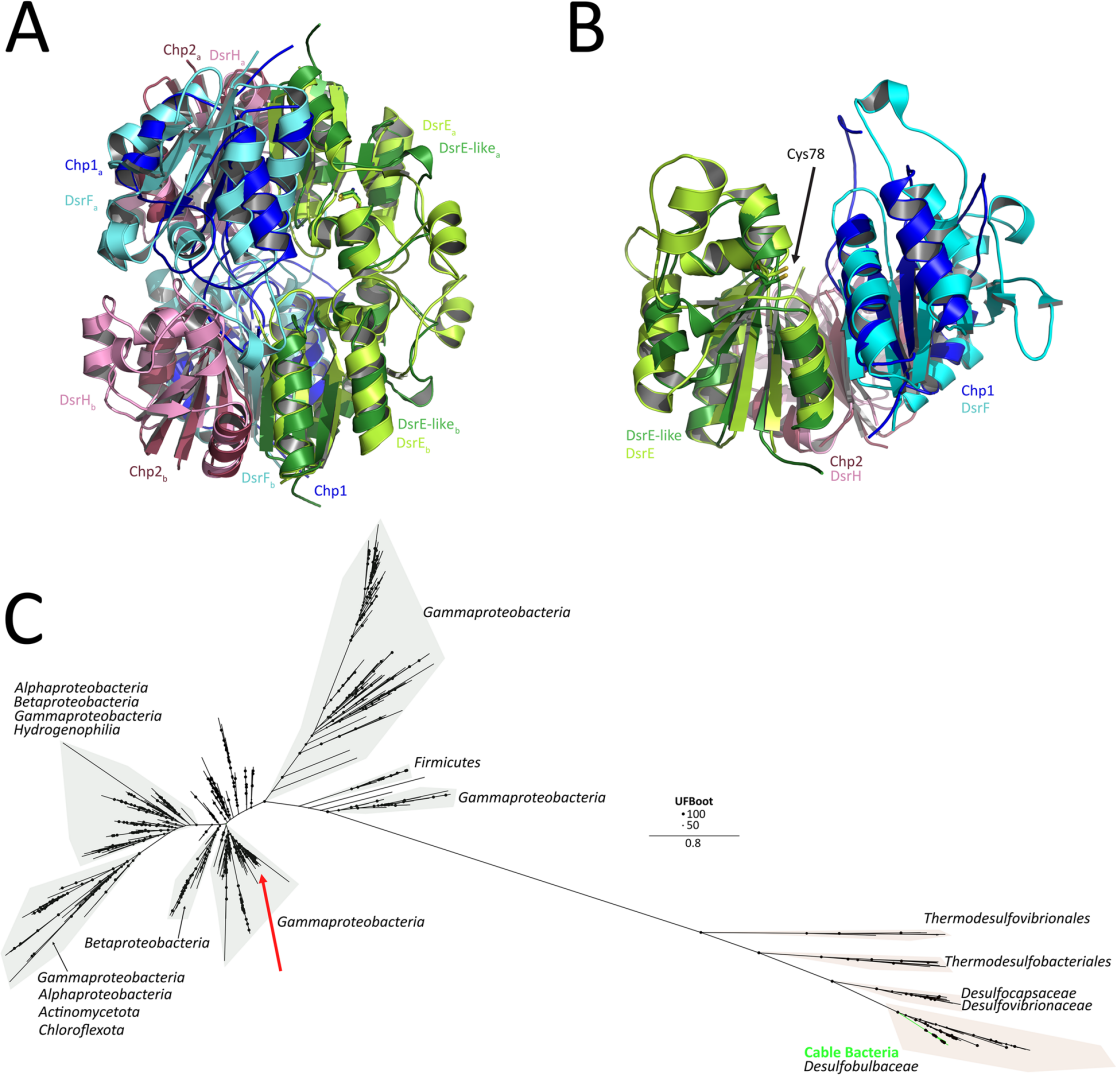
Analysis of the DsrE-like, Chp1/DsrF-like and Chp2/DsrH-like proteins of the YTD-cluster. **A** Superimposed DsrEFH from *A. vinosum* to the multimeric structural prediction using Alphafold2. DsrE-like, Chp1, Chp2 from *Ca*. Electrothrix scaldis GW3-3 are represented in darker colours, DsrEFH from *A. vinosum* in lighter colours. **B** Zoom-in of the panel A). The conserved Cys78 is depicted in stick representation and indicated with an arrow. **C** Unrooted maximum-likelihood phylogeny (model LG + F+I+G4) of cable bacteria DsrE-like protein sequences, RefSeq database hits, and previously identified DsrE proteins [49]. The clade containing DsrE from *A. vinosum* is marked with the red arrow. Cable bacteria sequences are non-orthologous to DsrE and cluster with DsrE-like sequences of the *Desulfobulbaceae*

To investigate the remaining YTD proteins (DsrE-like, Chp1 and Chp2), we modelled their structures using AlphaFold2 and compared them to the double heterotrimer DsrEFH of *Allochromatium vinosum* (PDB 2HY5). In *A. vinosum*, DsrEFH participates in a complex sulphur-trafficking network [6], transferring sulphur to DsrC, and the DsrC-bound-sulphur is subsequently used as a substrate by rDsrAB in the rDsr pathway [5, 81]. The predicted DsrE-like protein of cable bacteria shows strong structural similarity to *A. vinosum* DsrE (Z-score 12.4, RMSD 1.9 Å), including the conservation of the catalytic cysteine Cys78 [6] (Fig. 3A, B). The modelled Chp1 (DsrF-like) and Chp2 (DsrH-like) proteins align with DsrF (Z-score 6.9, RMSD 2.7 Å) and DsrH (Z-score 4.7, RMSD 2.7 Å), respectively, indicating an overall DsrEFH-like architecture (Fig. 3A). However, the DsrH-like proteins lack the conserved Cys20 residue essential for sulphur oxidation in *A. vinosum* [6]. Sequence-based alignments and phylogenetic analyses further reveal that these proteins are extremely divergent from canonical DsrEFH sequences (DsrE: Fig. 3C; DsrF-like and DsrH-like sequences from cable bacteria do not form proper sequence alignments with canonical DsrF and DsrH sequences).

Taken together, these findings suggest that the YTD-encoded DsrEFH-like complex is not orthologous to DsrEFH, but resembles a structural analogue, which could imply a related role in sulphur trafficking or sulphurylation. It is plausible that this complex interacts with sulphur species produced by the YeeE–YeeD system (sulphite and sulphide), as they are present on the same locus. Transfer of sulphur species other than sulphide is not unprecedented, as the sulphur-oxidising archaeon *Metallosphaera cuprina* employs a DsrE homologue for thiosulphate rather than sulphide trafficking [82]. The presence of this DsrEFH-like system in cable bacteria and other non-sulphide-oxidising *Desulfobulbaceae*, as well as its upregulation under sulphur disproportionation conditions in *Dl. dissulfuricans* [30] support a putative role in sulphur disproportionation reactions. Further biochemical characterisation will be essential to clarify its precise functional role and chemical binding properties.

### Cable bacteria encode a novel subtype of SQR and a rhodanese-PDO-like fusion protein

Beyond the Dsr pathway, cable bacteria encode an extensive repertoire of sulphur- metabolism genes (Fig. 1A). Some of these genes were previously identified [34, 35], but our analyses yielded slightly revised annotations.

A complete polysulphide/thiosulphate reductase (Phs/PsrABC) complex is present in all closed cable bacteria genomes, with subunits missing only in incomplete assemblies, thus indicating that this full complex is most likely universal within cable bacteria (Fig. 1A). Since the Phs and Psr subunits share high sequence and domain similarity, and because some characterized proteins exhibit overlapping substrate ranges - such as the ability of *Shewanella oneidensis* PsrABC to reduce both thiosulfate and elemental sulphur [83]- substrate specificity cannot be inferred from sequence alone [84]. Closely related *Desulfobulbaceae* encode only Phs/PsrAB and lack the membrane-bound NrfD-like Phs/PsrC subunit, possibly indicating a loss of quinone interaction [30]. Phylogenetic analysis further shows that the catalytic PsrA/PhsA of cable bacteria clusters with homologues from the *Thermodesulfovibrio* genus (Fig. S20), organisms capable of reducing sulphate, sulphite, and thiosulphate but not elemental sulphur. Therefore, their complex is considered a thiosulphate-specific reductase [85, 86]. This phylogenetic affiliation outside their phylum, combined with the consistent presence of the quinone-interacting Phs/PsrC, suggests a genetic adaptation in cable bacteria compared to other *Desulfobulbaceae* genera that may be linked to sulphur-related redox processes, although its physiological significance remains unclear.

Cable bacteria encode multiple rhodanese domain–containing proteins (Fig. 1A), with up to seven copies per genome depending on the species. These proteins are predicted to localize to either the cytoplasm or the periplasm (Table S2). Other *Desulfobulbales* also harbour both periplasmic and cytoplasmic rhodanese proteins (Fig. 1A). Phylogenetic analyses reveal that rhodanese sequences are not monophyletic and form distinct clades (Fig. S21), two of which are present in all cable bacteria genomes (Table S2; Fig. 1A). One is predicted to be cytoplasmic (cRho) and the other periplasmic (pRho), indicating that sulphur trafficking in cable bacteria likely occurs in both cellular compartments, and is not exclusively restricted to the cytoplasm as previously proposed [34].

**Fig. 4:**
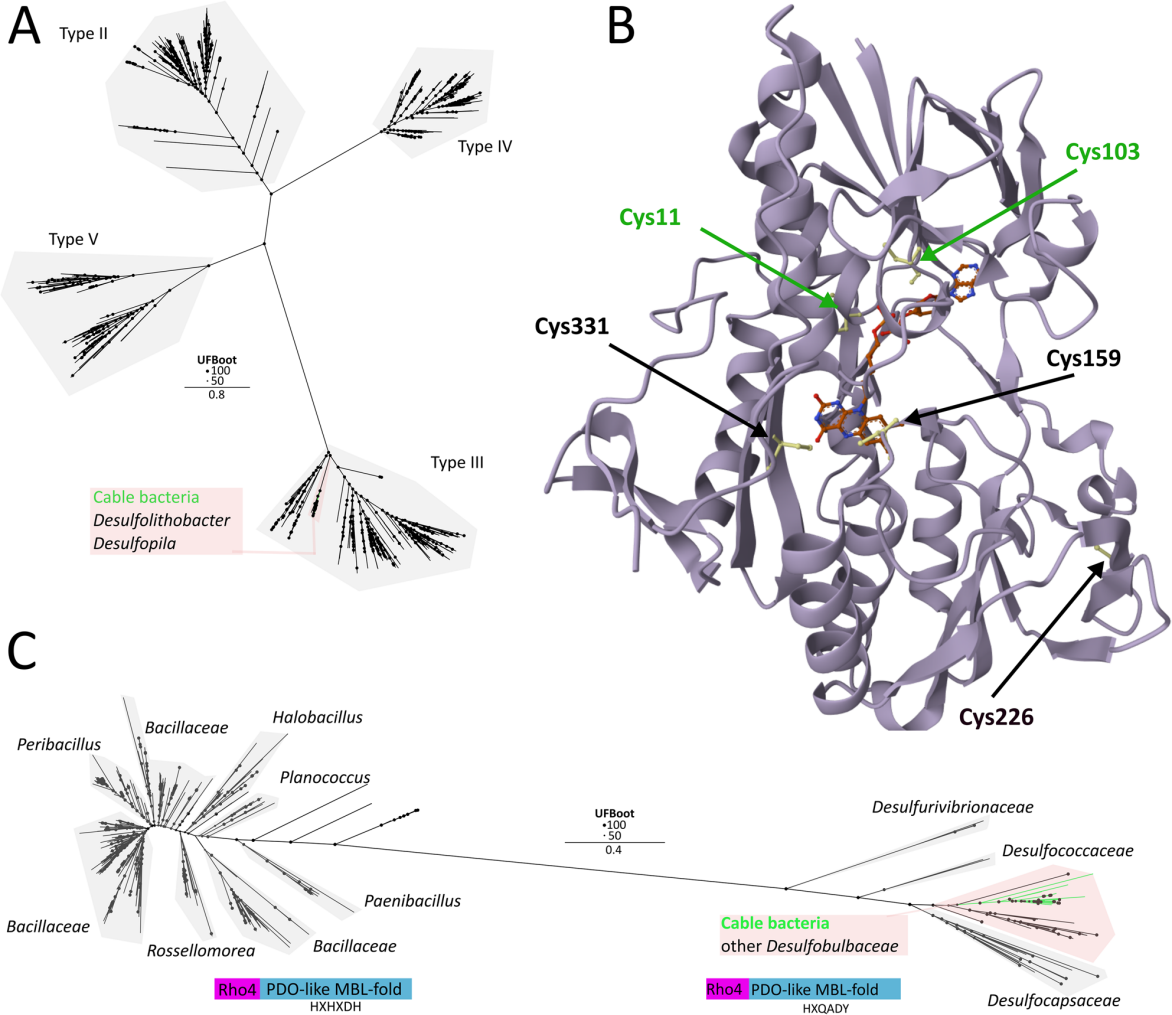
Analysis of the putative cable bacteria SQR and Rho-PDO. **A** Unrooted maximum-likelihood phylogeny (model Q.pfam + I+R10) of cable bacteria (green) and *Desulfobulbales* SQR sequences and database hits. Cable bacteria sequences form a subclade with sequences of the *Desulfolithobacter* and *Desulfopila* genera (red) within the larger SQR type III clade. Other *Desulfobulbales* sequences form a cluster with type II, IV and V SQRs. **B** *Ca*. Electrothrix scaldis GW3-3 SQR protein model, generated with Alphafold3. Cysteine residues are shown with arrows. Green cysteine residues are unique to the subclade seen in A, as determined by multiple sequence alignment (Fig. S19), and Cys11 and Cys103 are located in the solvent-accessible cavity. The FAD cofactor is indicated in red. **C** Unrooted maximum-likelihood phylogeny (model Q.yeast+R8) of cable bacteria (green) and *Desulfobulbales* Rho-PDO sequences and database hits. There is a clear separation between *Desulfobulbales* sequences (right) and *Bacillota* sequences (left). Both clades contain a Rho4 domain fused to a PDO-like MBL-fold domain, but differ in their metal-binding motif (indicated in figure)

Cable bacteria universally encode a putative sulphide:quinone oxidoreductase (SQR), an enzyme that catalyses the oxidation of sulphide to polysulphide coupled to quinone reduction via a flavin adenine dinucleotide (FAD) cofactor [87–89]. Polysulphides form on redox active cysteine pairs within a solvent-exposed cavity [90]. Previous models placed SQR in the periplasm [34, 35], yet our SignalP 6.0 analysis revealed no signal peptide [53], suggesting it is anchored to the inner membrane from the cytoplasmic side. This implies that elemental sulphur is created in the cytoplasm, and so it might be disconnected from periplasmic Psr/Phs activity. SQR homologues are also present in several *Desulfobulbales*, though absent from *Desulfobulbus* and *Desulfogranum* (Fig. 1). The SQR of *Dl. dissulfuricans* shares a high sequence identity with cable bacteria SQR (78%) and is also predicted to be cytoplasmic (Fig. 1A). Phylogenetic analysis and comparison with class-specific sequence fingerprints [91] place cable bacteria SQR within class III, forming a distinct subclade with sequences from *Dl. dissulfuricans* and two *Desulfopila* species (Fig. 4A). Multiple sequence alignment identified five conserved cysteine residues in cable bacteria SQRs (Cys11, Cys103, Cys159, Cys226, Cys339; based on *Ca.* E. scaldis GW3-3; Fig. S22). Cys159 and Cys339 correspond to the canonical catalytic cysteines of type III SQRs [91], whereas Cys11, Cys103, and Cys226 appear unique to cable bacteria (Fig. S22). Structural modelling (AlphaFold3; Fig. 4B) indicates that Cys11 and Cys103, together with the canonical Cys159 and Cys339, are located within the catalytic cavity adjacent to the FAD-binding site [88, 92]. In contrast, Cys226 is positioned away from known catalytic or FAD-interacting regions. The presence of additional thiols in the active-site pocket could suggest an expanded capacity for polysulphide formation. We therefore propose that the SQRs of *Ca.* Electronema, *Ca.* Electrothrix, *Desulfolithobacter*, and *Desulfopila* could represent a distinct subtype within class III SQRs, and likely play a central role in elemental sulphur/polysulphide formation.

Genomes encoding SQR and rhodanese often also contain a persulphide dioxygenase (PDO), which in *Cupriavidus pinatubonensis* acts together with SQR and rhodanese to oxidize sulphide to sulphite via glutathione persulphide (GSSH) intermediates [10, 93]. In cable bacteria, a putative PDO domain (InterPro: PDO-like MBL fold) is fused to an N-terminal rhodanese (Rho4) domain (Fig. 4C). Although Rho–PDO fusions have been reported previously [8], they remain uncharacterized, unlike the biochemically studied SQR–rhodanese fusion proteins [93]. The Rho–PDO fusion is present in all high-quality *Ca.* Electrothrix genomes but only in one *Ca.* Electronema genome (*Ca.* En. halotolerans), and thus not universal among cable bacteria (Fig. 1A). Rho-PDO homologues are also present in related *Desulfobulbales* (Fig. 1A), pointing to a possible gene loss in *Ca.* Electronema. Phylogenetically, cable bacteria and *Desulfobulbales* Rho–PDO sequences are closely related, and are distinct from *Bacillota* Rho–PDOs, which are largely found in aerobic or facultative anaerobes, whereas *Desulfobulbales* are mostly strict anaerobes (Fig. 4C). Note that overexpression of SQR under anaerobic conditions correlates with the expression of PDO in *Acidithiobacillus ferrooxidans*, while promoting sulphide oxidation to sulphite, suggesting that PDO does not need oxygen to function [94]. PDOs belong to the metallo-β-lactamase superfamily [95] and evolved from hydrolytic enzymes, sharing structural similarity with glyoxalase II [96]. They typically coordinate a single metal centre, often Fe²⁺, through two conserved histidines and an aspartic acid, also conserved in the modelled (Alphafold3) structure of cable bacteria PDO (Fig. S23). Canonical bacterial PDOs feature a conserved HXHXDH motif, where the first histidine ligates the metal centre and the remaining residues form a secondary coordination sphere that stabilizes catalysis [96]. This motif is conserved in *Bacillota* (Fig. 4C). In contrast, the PDOs in cable bacteria and *Desulfobulbales* lack the full motif, retaining only the first histidine (HXQADY; Fig. 4C), which likely still coordinates the single metal centre (Fig. S23). Although these PDOs are putatively catalytically active, their divergent motifs and distinct phylogenetic placement warrant further biochemical characterization to clarify their potential role in sulphite formation.

### Expression of sulphur metabolism genes in *Ca*. Electronema aureum and related organisms

**Fig. 5:**
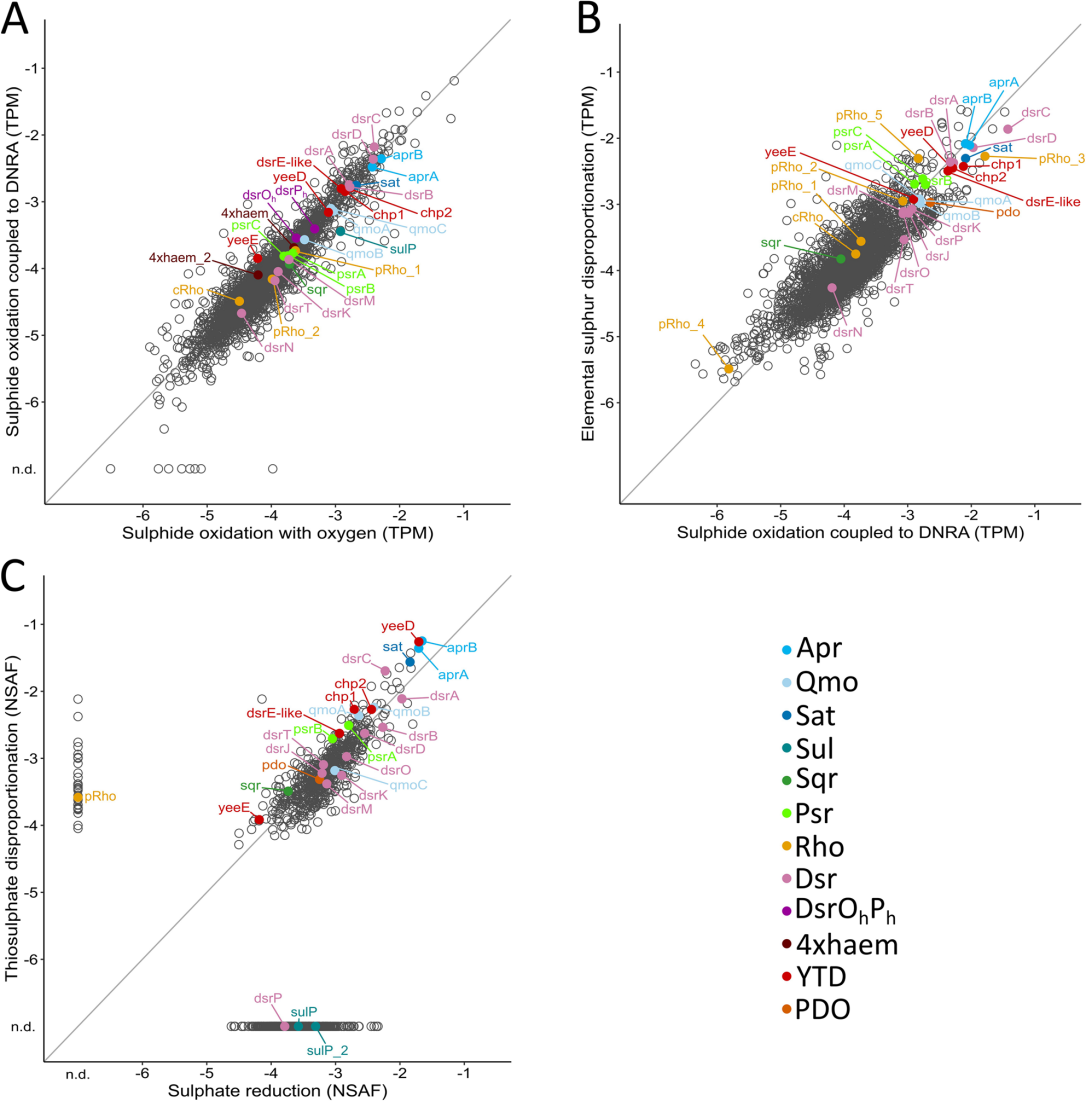
Analysis of expression of sulphur metabolism genes. **A** Gene expression in *Ca*. Electronema aureum grown in sulphide-oxidizing conditions coupled to oxygen reduction (x-axis) or DNRA (y-axis). **B** Gene expression in *Dv. alkaliphilus* growing with sulphide coupled to DNRA (x-axis) or by elemental sulphur disproportionation (y-axis). (**A**) and (**B**) Metatranscriptomics data were obtained from Kjeldsen et al. [34], Marzocchi et al. [64] and Thorup et al. [33] and reanalysed. **C** Proteins detected in *Dl. dissulfuricans* grown by sulphate reduction (x-axis) or by thiosulphate disproportionation (y-axis). Data were obtained from Hashimoto et al. [30] and replotted. Selected genes/proteins putatively involved in sulphur metabolism are indicated with coloured markers

Reanalysis of transcriptomic data of *Ca.* Electronema aureum under oxic [34] and nitrate-reducing [64] conditions shows that all genes implicated in sulphur metabolism (Fig. 1A) are expressed (Fig. 5A). Gene expression was highly similar across both conditions, indicating limited impact of the specific electron acceptor (O_2_ or NO_3_^−^) on sulphur metabolism. Notably, *dsrC*, *dsrD*, and *aprAB* were the most highly expressed genes. The Dsr pathway genes *dsrAB*, *dsrTMK*, *dsrN* were expressed in decreasing order, along with genes of the *dsrO*_*h*_*P*_*h*_-tetrahaem cluster (note that *dsrJ* is missing from the annotated genome; Table S2). The first tetrahaem gene in this cluster (universal to all cable bacteria; Fig. 1A) showed higher expression than the second (unique to *Ca.* En. Aureum; Table S2). Also expressed were *sat*, *qmoABC*, and *sulP*, as well as *sqr* and *psr/phsABC*, which displayed nearly identical expression levels. Periplasmic rhodaneses (pRho) were more highly expressed than cytoplasmic ones (cRho). All five genes of the YTD cluster were expressed, with the *dsrEFH*-like complex showing the highest expression, followed by *yeeD* and *yeeE*.

In *Dv. alkaliphilus*, genes of the Dsr pathway are highly expressed during sulphide oxidation coupled to DNRA and under sulphur disproportionation conditions [33]. The overall expression pattern in *Dv. alkaliphilus* is similar to *Ca.* En. aureum, though differences exist: *dsrC* is again most highly expressed, followed by *aprAB*, *dsrD*, *sat*, and *dsrAB* (Fig. 5B). Periplasmic rhodaneses and YTD genes are also strongly expressed, with nearly equal expression of *dsrEFH* and *yeeD*. Most sulphur metabolism genes rank among the top 250 expressed genes, including *pdo*. Lower expression was observed for *cRho*, *dsrT*, *dsrN*, and *sqr* (Fig. 5B). Unlike *Ca.* En. aureum, *Dv. alkaliphilus* shows very high expression of two *pRho* genes but low *sqr* expression. The *Dv. alkaliphilus* SQR lacks a signal peptide (SignalP 6.0) and is thus predicted to localize on the cytoplasmic side of the membrane, as is the case in cable bacteria.

Since *Dv. alkaliphilus* cannot grow by sulphate reduction, we further compared gene expression (proteomics data) under thiosulphate disproportionation and sulphate reducing conditions in *Dl. dissulfuricans* [30]. Under disproportionating conditions, YTD proteins were more abundant than under sulphate reduction (Fig. 5C). YeeD, AprAB, SAT, DsrC, DsrAB, and PDO were highly expressed. All DsrMKJOP complex proteins were detected. No expression was detected for the DsrO_h_P_h_-tetrahaem proteins. A pRho protein was detected only under thiosulphate disproportionation conditions (Fig. 5C).

Overall, the expression profiles across the three organisms show a similar sulphur metabolism gene repertoire active under diverse conditions. Beyond the canonical *dsr*, *apr*, and *qmo* genes, the YTD cluster is consistently and highly expressed (Fig. 5), supporting its role in sulphur disproportionation [51, 78] and suggesting sulphur relay by the DsrEFH-like complex (DsrE-like, Chp1 and Chp2; Fig. 1A). Compared to the other analysed *Desulfobulbales*, *Ca.* En. aureum shows much higher *sqr* and *psr/phsABC* expression and also expresses the *dsrO*_*h*_*P*_*h*_*-tetrahaem* genes that are not expressed in *Dl. dissulfuricans*.

### A model for the electrogenic sulphur metabolism in cable bacteria

The key physiological innovation distinguishing cable bacteria from their relatives is the capability of long-distance electron transport [23], which enables the coupling of “anodic” sulphide oxidation in deeper sediment layers to “cathodic” oxygen reduction near the sediment-water interface. The process of anodic sulphide oxidation can be summarized by the reaction:

$$\:{\mathbf{H}\mathbf{S}}^{-}+4\:{\mathbf{H}}_{2}\mathbf{O}\to\:\:{{\mathbf{S}\mathbf{O}}_{4}}^{2-}+9\:{\mathbf{H}}^{+}+8\:{\mathbf{e}}^{-}$$

The electrons resulting from sulphide oxidation are channelled towards the conductive fibre system in the cell envelope, which transports them upwards towards the oxic zone, where cathodic oxygen reduction takes place:

$$\:2\:{\mathbf{O}}_{2}+8\:{\mathbf{H}}^{+}+\:8\:{\mathbf{e}}^{-}\:\to\:4\:{\mathbf{H}}_{2}\mathbf{O}$$

As such, the conductive fibre system *de facto* acts as the electron acceptor. As a result, the overall reaction is:

$$\:{\mathbf{H}\mathbf{S}}^{-}+2\:{\mathbf{O}}_{2}\:\to\:\:{{\mathbf{S}\mathbf{O}}_{4}}^{2-}+{\mathbf{H}}^{+}$$

**Fig. 6:**
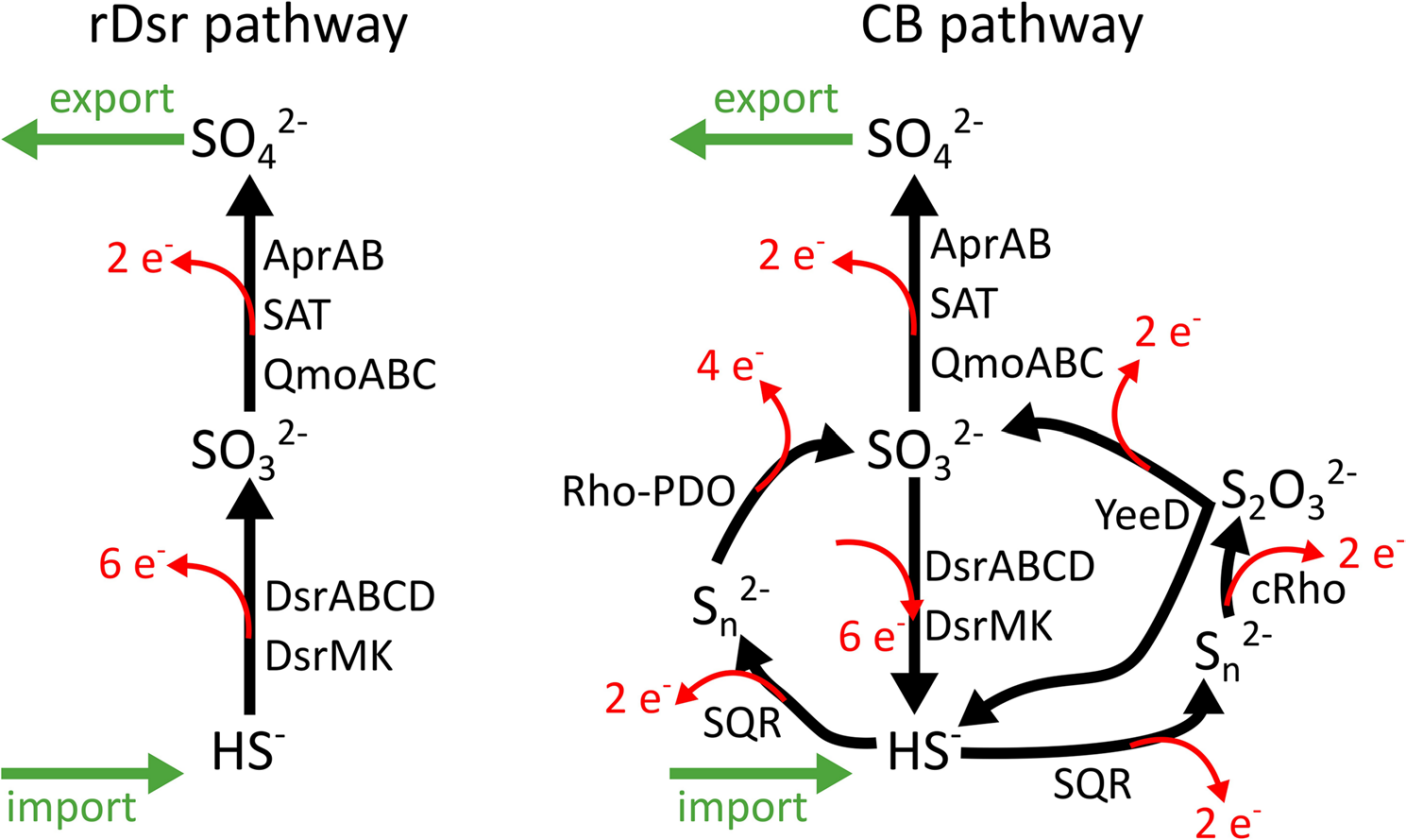
Two possible models for net sulphide oxidation in cable bacteria. **A** the reversed Dsr pathway as proposed in Kjeldsen et al. [34] (**B**) The novel “CB pathway” as proposed here

The metabolism of cable bacteria hence results in a net oxidation of sulphide (HS^−^) to sulphate (SO_4_^2−^), as testified by the characteristic sulphide depletion and sulphate accumulation observed in the deeper part of the sediment colonized by cable bacteria [15, 97]. Previously it has been proposed that this “anodic” sulphide oxidation reaction runs through a reversal of the canonical sulphite reductase DsrAB in the Dsr pathway [34] (Fig. 6). Yet, as shown in our analysis, an important obstacle for this proposed pathway is that the DsrABCD system found in cable bacteria is of the reductive type. Such reductive-type DsrABCD systems are considered biochemically implausible to operate in reverse [37, 38], making sulphide oxidation to sulphite via this enzyme cascade unlikely. Indeed, to the best of our knowledge, no experimental evidence for the reversal of a reductive type DsrAB has ever been shown. By contrast, other components of the reductive Dsr pathway (i.e. AprAB-QmoABC-SAT cascade) have been proposed to be reversible and may catalyze the oxidation of sulphite to sulphate under oxidizing conditions [98–102]. Thus, in cable bacteria, we propose that the Dsr pathway most plausibly involves disproportionation at the level of sulphite, with oxidation to sulphate via SAT–AprAB–QmoABC, and reduction to sulphide via the reductive DsrABCD-DsrMKJ system (Fig. 6).

Several lines of evidence from our results support this idea: (1) the phylogenetic affiliation of DsrABCDMKJ in cable bacteria with reductive-type proteins of other *Desulfobulbaceae* (Fig. S2-4, S6); (2) the consistent presence and expression of *dsrD* (Fig. 1A, A), that is only found in sulphur reduction or disproportionation metabolisms [37, 38]; (3) the absence a canonical DsrL protein [67], which are considered essential in the reverse (rDsr) pathway [5]; and (4) the overall similarity in genetic repertoires and expression levels of sulphur metabolism genes in cable bacteria and related *Desulfobulbales* (Fig. 5).

Instead, we propose that cable bacteria oxidize sulphide through a mechanism analogous to that used by sulphur-disproportionating *Desulfobulbaceae*. Early studies on the closely related *Desulfobulbus (Db.) propionicus* [103] and other sulphate reducers (e.g. *Desulfovibrio desulfuricans*) [104] demonstrated that when these organisms are exposed to oxygen and sulphide, the first detectable step is the formation of elemental sulphur/polysulphides:

$$\:4\:{\mathbf{H}\mathbf{S}}^{-}+2\:{\mathbf{O}}_{2}+4\:{\mathbf{H}}^{+}\:\to\:\:{{\mathbf{S}}_{\mathbf{n}}}^{2-}+4{\mathbf{H}}_{2}\mathbf{O}$$

This reaction requires an external electron acceptor (oxygen). The resulting polysulphides are then disproportionated in an electron-neutral step:

$$\:{{\mathbf{S}}_{\mathbf{n}}}^{2-}+4\:{\mathbf{H}}_{2}\mathbf{O}\:\to\:\:{{\mathbf{S}\mathbf{O}}_{4}}^{2-}+3\:{\mathbf{H}\mathbf{S}}^{-}+5\:{\mathbf{H}}^{+}$$

Experimental work indicates that sulphite is likely produced as a transient intermediate during this disproportionation [99, 103], although the exact enzymatic steps remain unresolved. The combined stoichiometry of the oxidative and disproportionation steps is:

$$\:{\mathbf{H}\mathbf{S}}^{-}+2\:{\mathbf{O}}_{2}\:\to\:\:{{\mathbf{S}\mathbf{O}}_{4}}^{2-}+{\mathbf{H}}^{+}$$

This same net transformation is observed in cable bacteria. Importantly, this pathway is mechanistically distinct from a reversal of the Dsr system. It requires formation and turnover of elemental sulphur/polysulphides as obligate intermediates [103], rather than direct oxidation of sulphide to sulphite via reverse DsrAB [5]. We therefore propose that cable bacteria perform sulphide oxidation via a more intricate metabolic configuration that draws on enzyme systems broadly conserved across *Desulfobulbaceae* (Fig. 1). However, a key distinction between cable bacteria and other *Desulfobulbaceae* is that the sulphide oxidation and oxygen reduction half-reactions occur in separate cells, which is facilitated by long-distance electron transport. We refer to this configuration as the “cable bacteria (CB) pathway”, with its core transformations and electron flows summarized in Fig. 6.

The CB pathway combines a set of sulphur metabolism enzyme systems (Fig. 6). Just like in the proposed rDsr pathway, sulphite (SO_3_^2−^) is a key intermediate, and is oxidized to sulphate (SO_4_^2−^) via the reversible AprAB-QmoABC-SAT branch. However, a crucial difference lies in the origin of SO_3_^2−^. In the CB pathway, SO₃²⁻ is not produced from HS⁻ via reverse DsrABCD (as we argue this reaction is not reversible). Instead, HS⁻ is oxidized to elemental sulphur/polysulphides (Sₙ²⁻) by SQR, and subsequently to SO_3_^2−^ via the Rho–PDO system (Fig. 6). A further distinguishing feature is the role of thiosulfate (S_2_O_3_^2−^), which can function as an additional intermediate in the conversion of S_n_^2−^ to SO_3_^2−^. Finally, cable bacteria express the DsrABCD complex at very high levels. We argue that this system operates in its conventional reductive direction, reducing SO_3_^2−^ back to HS⁻ (Fig. 6). Although this back-reaction appears to oppose anodic HS⁻ oxidation, an analogous phenomenon was reported in *Db. propionicus* under aerobic conditions [104]. We propose that this reductive flux may serve a regulatory function, potentially as an overflow mechanism that prevents the intracellular accumulation of SO_3_^2−^. The HS⁻ produced by this reductive flux can re-enter the pathway via SQR, a reaction which is sustained in cable bacteria through electrical linkage with cells in the oxic zone. Thus, ultimately, only SO_4_^2−^ accumulates as the net end product, consistent with the biogeochemical observations in sediments inhabited by cable bacteria.

Thus, the CB pathway exhibits a “disproportionation” character, whereby certain sulphur compounds (particularly SO_3_^2−^and S_2_O_3_^2^) are simultaneously oxidized and reduced. Previous work has speculated that a disproportionation-type sulphur cycle occurs in cable bacteria [37, 38], although the underlying molecular mechanism remains undefined. A similar metabolism has been proposed for the CB1MN strain, closely related to *Dv. alkaliphilus* [32]. In *Dv. alkaliphilus*, HS^−^ oxidation proceeds via S_n_^2−^ intermediates [33], consistent with the mechanism we propose here. Furthermore, *Dl. dissulfuricans*, a chemolithoautotroph from anoxic hydrothermal vents, can grow either as an H₂-dependent SO_4_^2−^reducer or via disproportionation of S_o_, S_2_O_3_^2^, and tetrathionate in the presence of ferrihydrite [30]. Finally, early observations with *Db. propionicus* indicate that it can oxidise HS^−^ to a S_n_^2−^, with subsequent disproportionation via a SO_3_^2−^ intermediate [103, 104]. Taken together, and considering the minimal genetic adaptation in cable bacteria compared to these relatives, our observations suggest that a genomic capacity for HS^−^ oxidation arose in the common ancestor of *Desulfobulbaceae*. Cable bacteria have further adapted this system to channel electrons from HS^−^ oxidation into their conductive network and can readily dispose of these electrons via their connection to oxygen. 

**Fig. 7:**
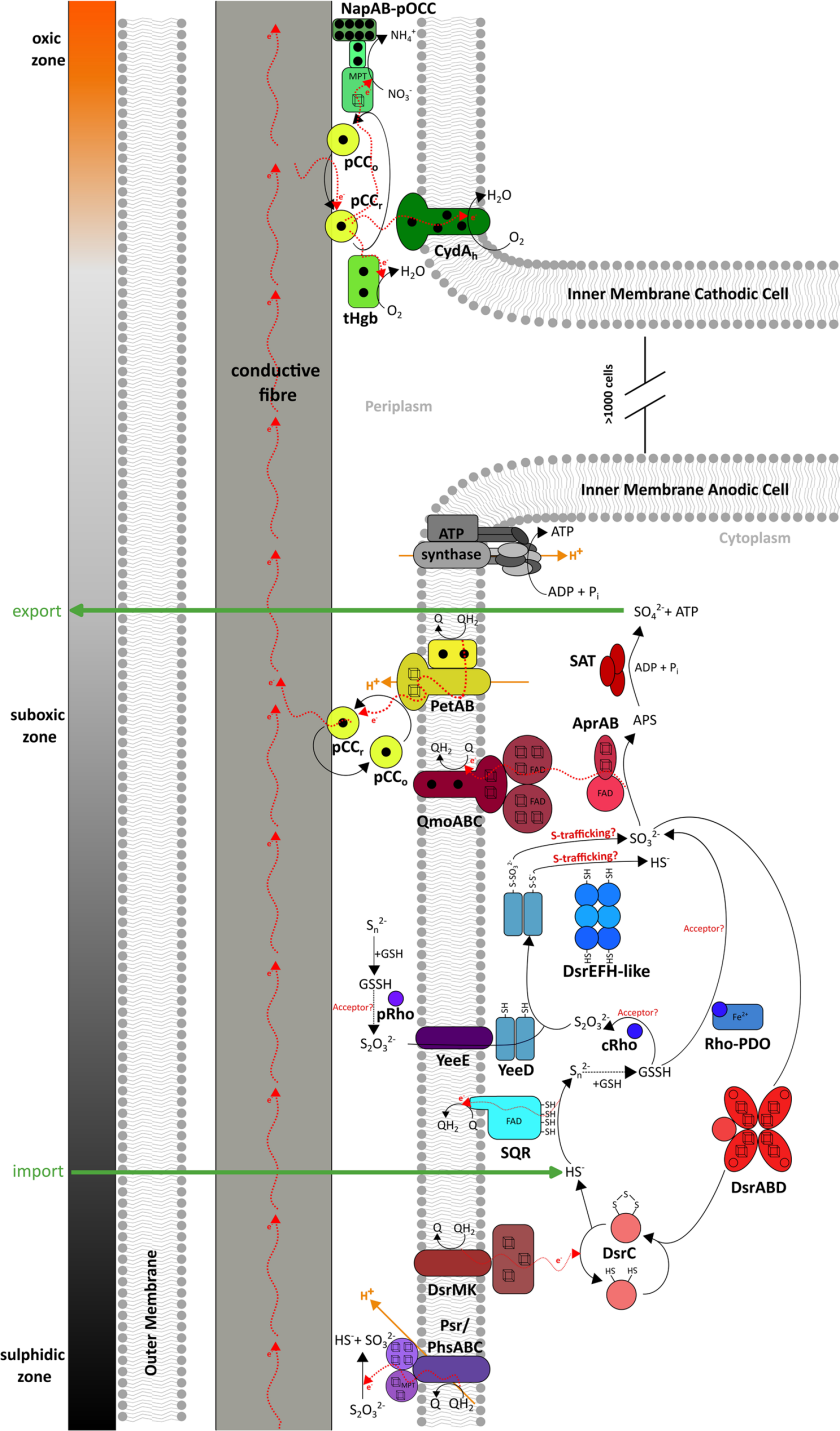
Schematic overview of proposed sulphur metabolism in cable bacteria. Putative proteins involved are indicated. Enzymatic reactions are shown with solid black arrows, spontaneous reactions with dashed black arrows. Electron flow (red arrows) and proton translocation (orange arrows) are indicated. Sulphur metabolism takes place in the sulphidic and suboxic zone. Sulphite generated through the putative SQR-mediated pathway (blue) is used for disproportionation by Dsr-pathway proteins (red). The overall reaction is being driven towards the production of sulphate, generating reduced menaquinone (QH_2_). Electrons are taken from the membrane pool and transferred to the periplasmic conductive fibre, putatively through the PetAB complex and pCC. In the oxic zone, electrons are taken from the periplasmic fibre and used for oxygen or nitrate reduction (green). Unknown reaction partners and speculative functions of proteins are indicated with red text

Figure 7 displays a more elaborate version of electrogenic S-oxidation metabolism in cable bacteria. In this model, HS^−^ is first oxidized by SQR at the cytoplasmic side of the membrane, producing S_n_^2−^ and reduced quinones [10, 88, 105]. S_n_^2−^ react with glutathione to form GSSH [10, 93], which can either be converted to SO_3_^2−^ by the Rho–PDO fusion protein [10, 96] or to S_2_O_3_^2−^ through rhodanese-mediated (cRho) reactions [93, 106], both requiring an as-yet-unidentified electron acceptor. Cytoplasmic thiosulphate may then be decomposed by the YTD-encoded [51] YeeD dimer, forming persulphide (-S-S^−^) and perthiosulphide (-S-SO_3_^2−^) intermediates on its catalytic cysteine residue [80]. In vitro, reducing conditions allow for the spontaneous release of HS^−^ and SO_3_^2−^, but in vivo, the release of these sulphur species is likely dependent on unknown acceptor proteins [80]. We speculate that the genetically adjacent DsrEFH-like complex encoded in the YTD cluster (Fig. 3A) could be this acceptor protein complex. This could further participate in S-trafficking, analogous to canonical DsrEFH in *A. vinosum* [6], ultimately leading to the release of free HS^−^ or SO_3_^2−^.

SO_3_^2−^ may be disproportionated into SO_4_^2−^ and HS^−^ via the canonical Dsr pathway, operating in opposite directions starting from SO_3_^2−^. SO_3_^2−^ reduction to HS^−^ may proceed through DsrABCD, with electrons supplied from DsrMK(J), which together constitute the minimal machinery required for SO_3_^2−^reduction [68]. The role of the TMH_1_-DsrO_h_P_h_–tetrahaem module remains elusive, but it may participate in alternative redox interactions or electron transport from the quinone pool to the periplasm. Nonetheless, its consistent expression in cable bacteria (Fig. 4C) warrants further biochemical characterization to elucidate its function. On the other side of the disproportionation reaction (Fig. 7), SO_3_^2−^ oxidation proceeds via AprAB–QmoABC to form adenosine-5′-phosphosulphate (APS), which is then converted to SO_4_^2−^ by SAT [98–100].

Cable bacteria potentially also utilize externally acquired S_n_^2−^ and/or S_2_O_3_^2−^, as suggested by their genomic potential and expression (Fig. 1, 4 A). The periplasmic Phs/PsrABC complex may reduce S_2_O_3_^2−^ to HS^−^ and SO_3_^2−^ or S_n_^2−^ to HS^−^, though its substrate range remains to be experimentally confirmed. Periplasmic elemental sulphur could also react with GSSH, with periplasmic rhodaneses (pRho) promoting thiosulphate formation [93]. S_2_O_3_^2−^ may then enter the cytoplasm via YeeE and be decomposed by YeeD [79, 80]. Collectively, these reactions likely integrate all reduced sulphur species (HS^−^, SO_3_^2−^, S_n_^2−^, and S_2_O_3_^2−^) into a unified CB pathway (Fig. 7) providing flexibility in sulphur species utilization.

Electrons generated from sulphur redox reactions will enter the quinone pool, and the electrons from the reduced quinones are most likely transferred to a partial complex III composed of a Rieske Fe–S protein and membrane-bound cytochrome *b* (PetAB) [34]. Electrons can be further transferred to periplasmic cytochrome *c* proteins [34], which can act as redox shuttles (pCC_r_ and pCC_o_; Fig. 7), ultimately transferring electrons to the conductive fibres [24, 25]. These fibres form an interconnected network linking all cells [22, 107]. In surface sediment layers, these electrons are ultimately used for oxygen reduction [77], potentially via truncated haemoglobin-pentahaem fusion proteins (tHgb-PHC) or the CydA-homologue (CydA_h_), which contains a periplasm-oriented cytochrome *c* domain. Periplasmic cytochromes could also be involved in O_2_ reduction, as observed in *Desulfovibrio* species [108, 109]. Alternatively, in some but not all cable bacteria species [35, 40, 41], electrons may drive nitrate reduction through the NapAB–pOCC complex [35, 64, 110].

## Conclusion

Overall, our comparative genomic analysis reveals that cable bacteria possess a genomic capacity for dissimilatory sulphur metabolism, with only minimal genetic divergence from closely related *Desulfobulbaceae*. They encode a complete reductive-type Dsr pathway, vertically inherited with no evidence of horizontal gene transfer, and harbour additional sulphur-metabolism genes: a novel sulphide: quinone oxidoreductase (SQR), a putative rhodanese–persulphide dioxygenase fusion (Rho–PDO), a *dsrOhPh–tetrahaem* gene cluster of unknown function, the Phs/PsrABC thiosulphate/polysulphide reductase complex, and a DsrEFH-like double trimer in the YTD cluster - all also present in their closest relative, *Dl. dissulfuricans*. Transcriptomic and proteomic reanalyses confirm expression of this full repertoire, with patterns largely paralleling those in *Dl. dissulfuricans* and *Dv. alkaliphilus*.

However, genome and expression data alone do not allow a complete reconstruction of the sulphide oxidation pathway. Several redox partners remain unidentified, and the roles of some genes remain speculative. Targeted biochemical and physiological studies are therefore needed to validate these functions and fully resolve the sulphur-metabolism network in cable bacteria.

Despite these uncertainties, our results challenge the assumption that cable bacteria oxidize sulphide via a reversed Dsr pathway. Instead, they support a disproportionation-based mechanism, in which elemental sulphur and sulphite act as central intermediates, and sulphide generated by disproportionation can re-enter the cycle. The key innovation appears to be the conductive periplasmic fibre network, which channels electrons from sulphide oxidation over long distances to oxygen. This suggests that conserved reductive-type sulphur pathways (typically associated with reduction or disproportionation in related organisms) possess substantial metabolic plasticity, which cable bacteria exploit to occupy novel ecological niches.

## Supplementary Information

Supplementary Material 1.

## Data Availability

Metatranscriptomic datasets for *Ca. Electronema aureum* (BioProject accessions: **PRJNA575166**, Kjeldsen et al. 2019; **PRJNA575156**, Marzocchi et al. 2022) and *Desulfurivibrio alkaliphilus* (accession: **PRJNA322753**, Thorup et al. 2017) are accessible through the National Center of Biotechnology Information (NCBI), Short Read Archive (SRA). Proteomic data for *Desulfolithobacter dissulfuricans* (Hashimoto et al. 2022) are available via the ProteomeXchange Consortium through the jPOST repository (accession: **JPST001840**). All genomes analysed in this study are publicly available through GenBank or RefSeq; accession numbers are provided in Supplementary Table S1.#### All protein sequences used as input for phylogenetic analyses and structural predictions in this study are available on Figshare (10.6084/m9.figshare.30869420).
