# Supplementary material for: The genetic repertoire underlying electrogenic sulphur oxidation in cable bacteria
Source: BMC Genomics. 2026 Mar 11;27:340. doi: 10.1186/s12864-026-12675-1 (PMC13047784; doi:10.1186/s12864-026-12675-1)
Supplement: Supplementary file 1 — Supplementary Material 1. [file 12864_2026_12675_MOESM1_ESM.pdf]

Supplementary information for:

The genetic repertoire underlying electrogenic sulphur oxidation in cable bacteria

Anwar Hiralal[1], Jeanine S. Geelhoed[1], Sinje Neukirchen[1,2], Val Karavaeva[2], Filipa L. Sousa[2] and Filip J. R. Meysman[1]

1. Geobiology Research Group, University of Antwerp, Antwerp, Belgium;
2. Department of Functional and Evolutionary Ecology, University of Vienna, Austria

Supplementary figures

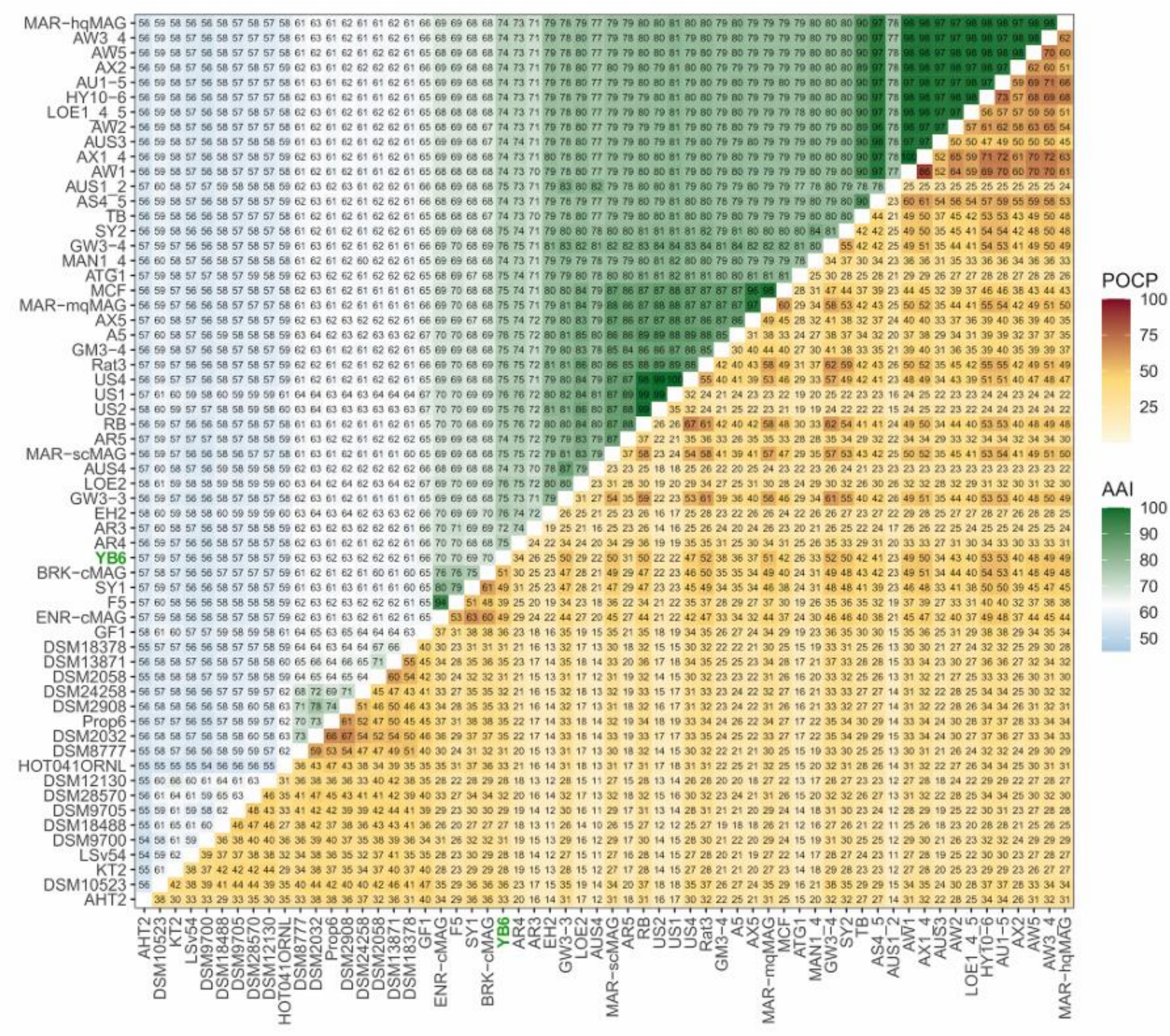

Figure S1: amino acid identity and percentage of conserved proteins table of cable bacteria and related species used in this study. Table is generated using a custom script provided by Sereika et al., 2023. Strain names can be found in table S1.

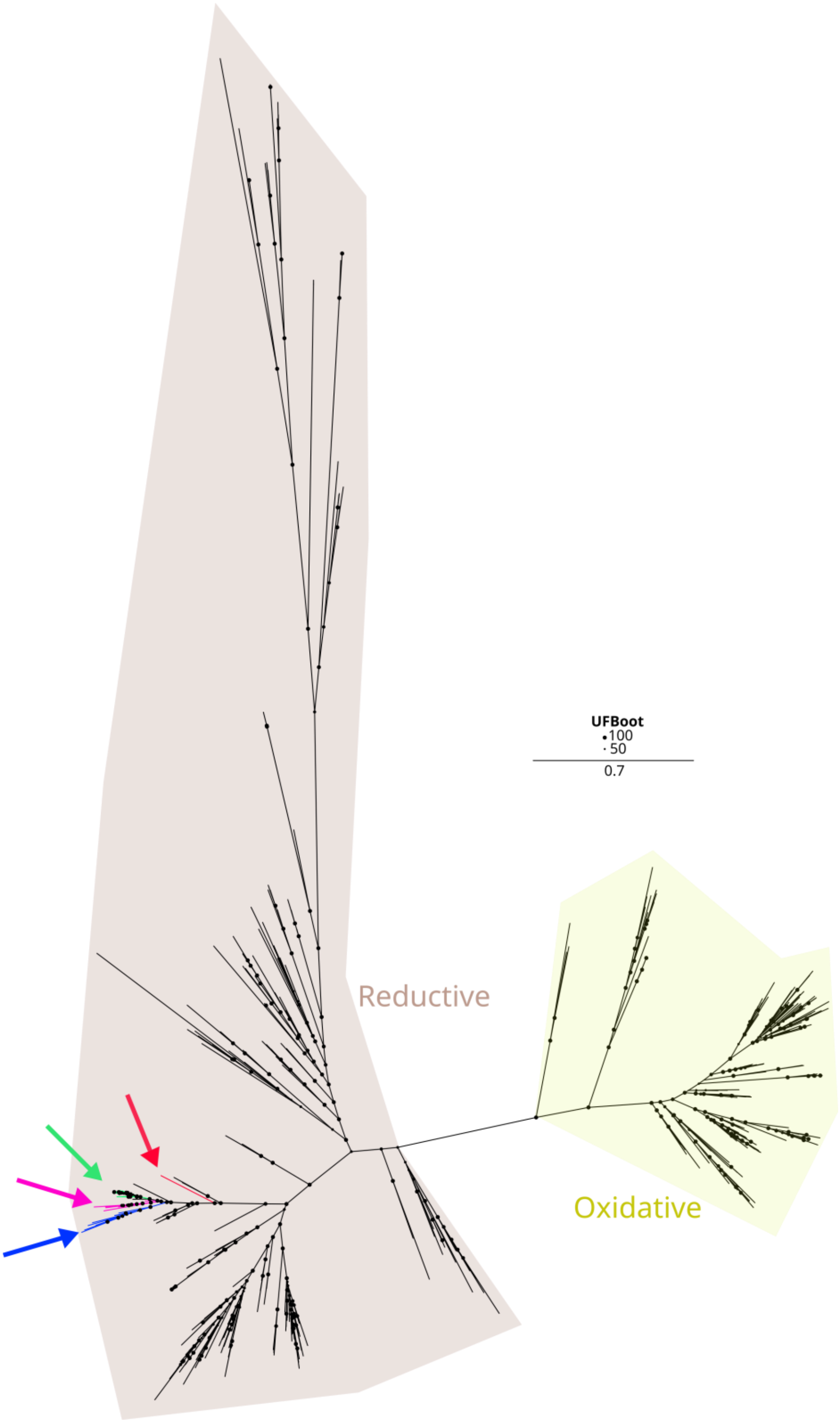

Figure S2: Maximum-likelihood phylogeny (unrooted) of cable bacteria- DsrA and reference sequences. Phylogeny inferred using IQTree (Nguyen et al., 2015) according to the best-fit model (model LG+I+R10). Reductive and oxidative branches are based on previously published data (Neukirchen and Sousa, 2021). Cable bacteria sequences are indicated (green), as well as other *Desulfobulbaceae* sequences (pink), *Desulfocapsaceae* sequences (blue) and *Desulfurivibrionaceae* sequences (red).

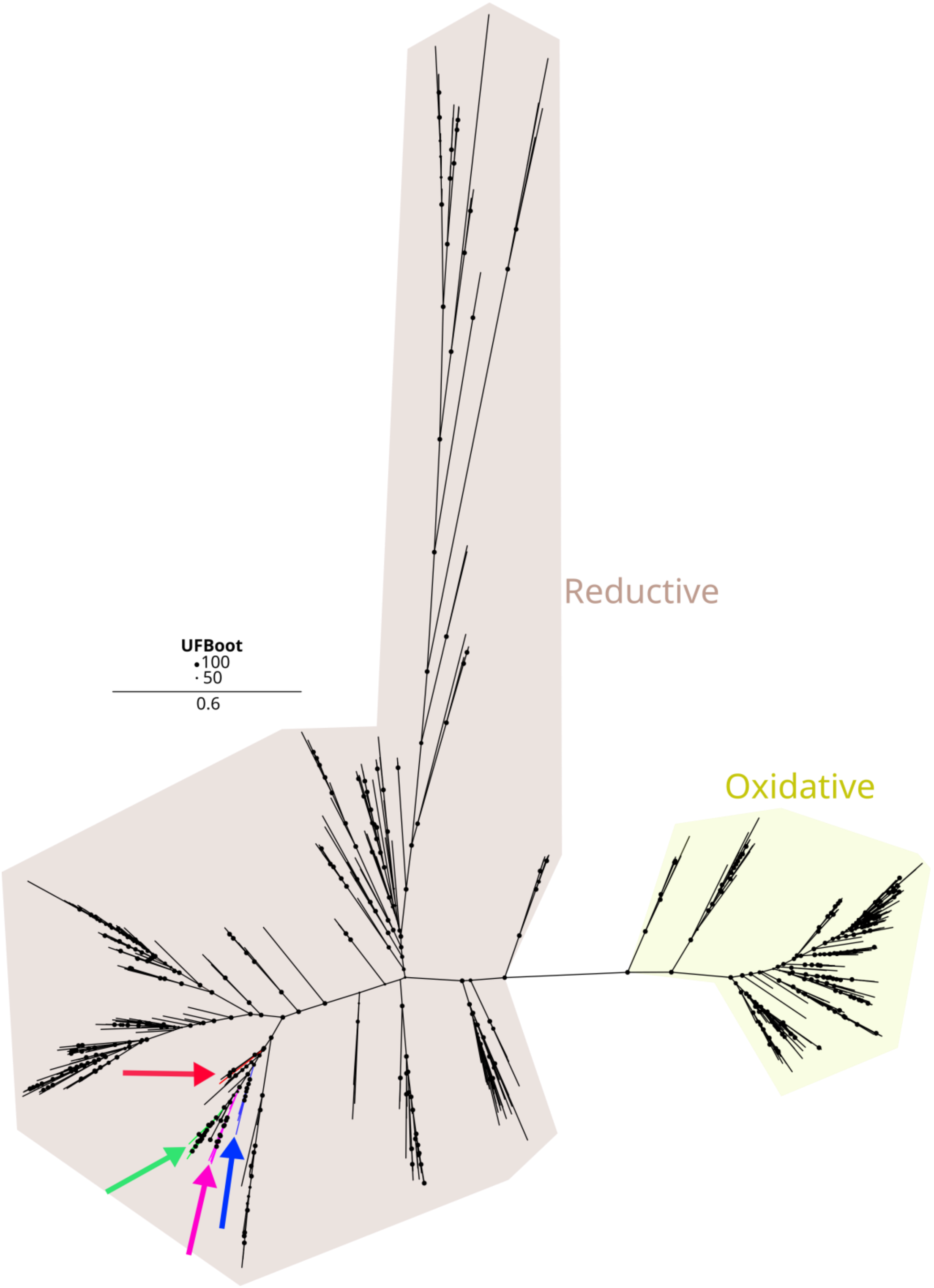

Figure S3: Maximum-likelihood phylogeny (unrooted) of cable bacteria DsrB and reference sequences. Phylogeny inferred using IQTree (Nguyen et al., 2015) according to the best-fit model (model LG+I+R10). Reductive and oxidative branches are based on previously published data (Neukirchen and Sousa, 2021). Cable bacteria sequences are indicated (green), as well as other *Desulfobulbaceae* sequences (pink), *Desulfocapsaceae* sequences (blue) and *Desulfurivibrionaceae* sequences (red).

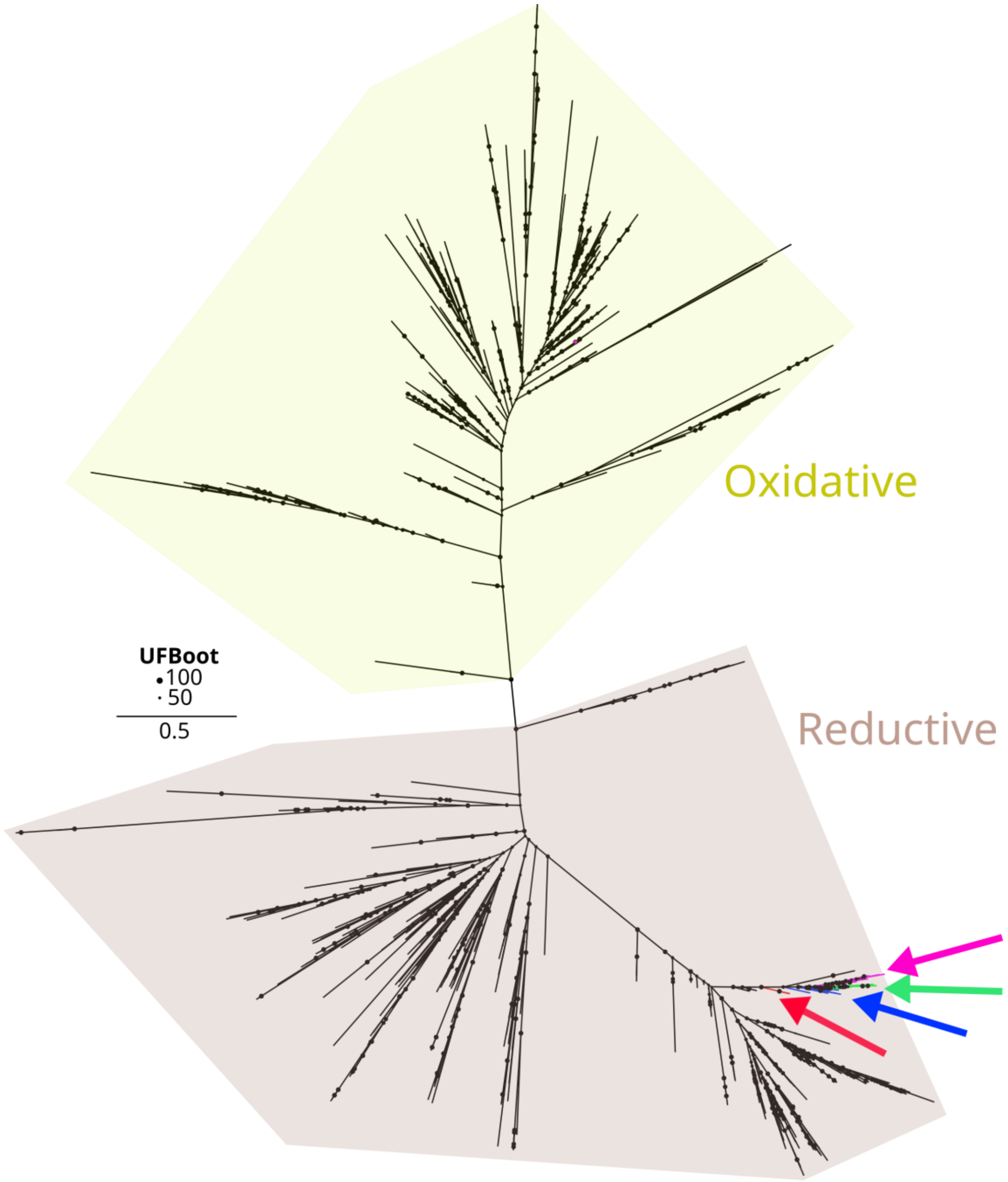

Figure S4: Maximum-likelihood phylogeny (unrooted) of cable bacteria DsrC and reference sequences. Phylogeny inferred using IQTree (Nguyen et al., 2015) according to the best-fit model (model Q.yeast+I+R9). Reductive and oxidative branches are based on previously published data (Neukirchen and Sousa, 2021. Cable bacteria sequences are indicated (green), as well as other *Desulfobulbaceae* sequences (pink), *Desulfocapsaceae* sequences (blue) and *Desulfurivibrionaceae* sequences (red).

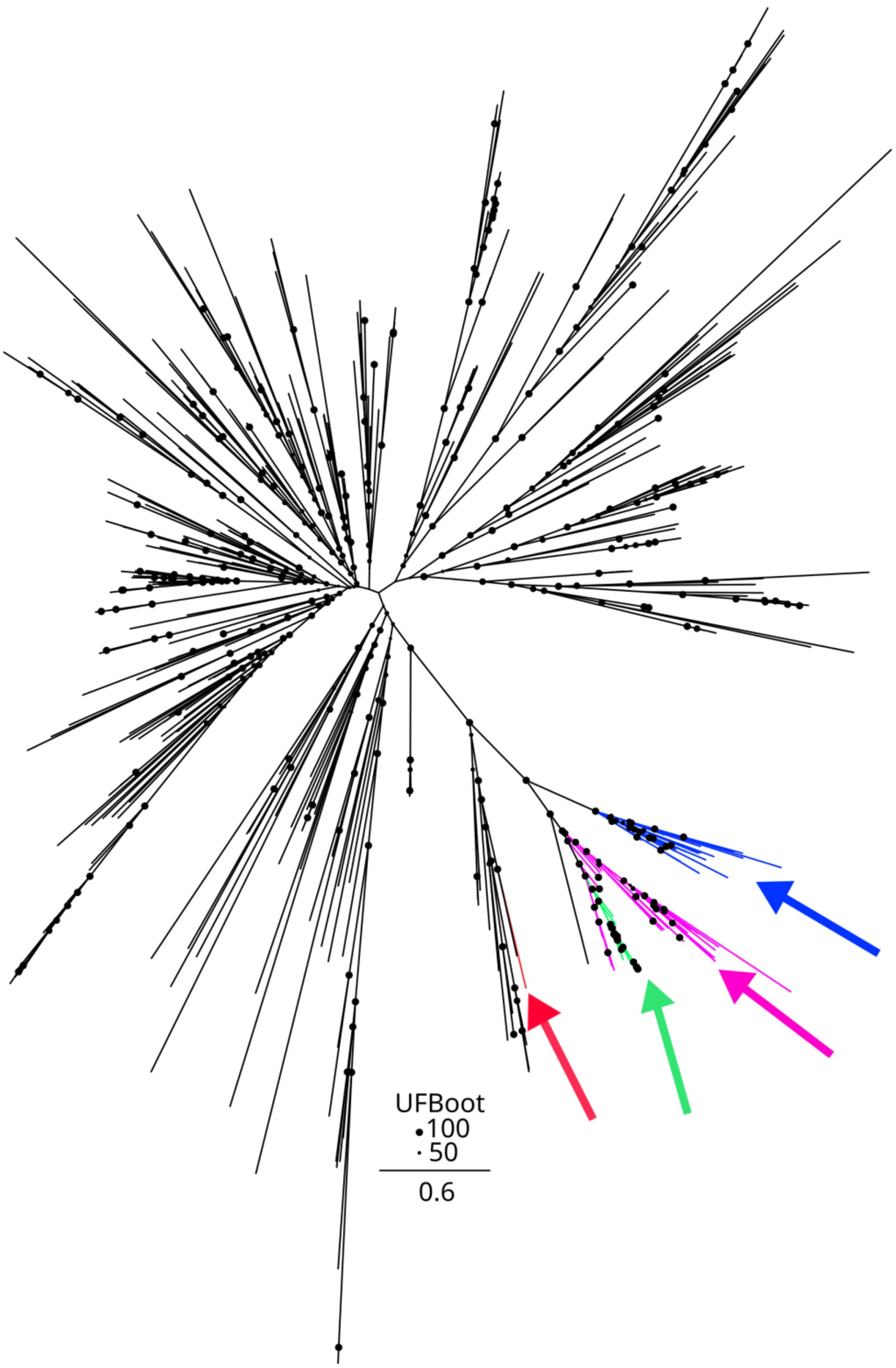

Figure S5: Maximum-likelihood phylogeny (unrooted) of cable bacteria DsrT and reference sequences. Phylogeny inferred using IQTree (Nguyen et al., 2015) according to the best-fit model (model Q.pfam+R8). Reference sequences are taken from a previously published study (Neukirchen and Sousa, 2021. Cable bacteria sequences (green) cluster with *Desulfobulbaceae* (pink) sequences. *Desulfocapsaceae* (blue) and *Desulfurivibrio alkaliphilus* (red) are more distantly related. DsrT sequences lack the oxidative type distinction.

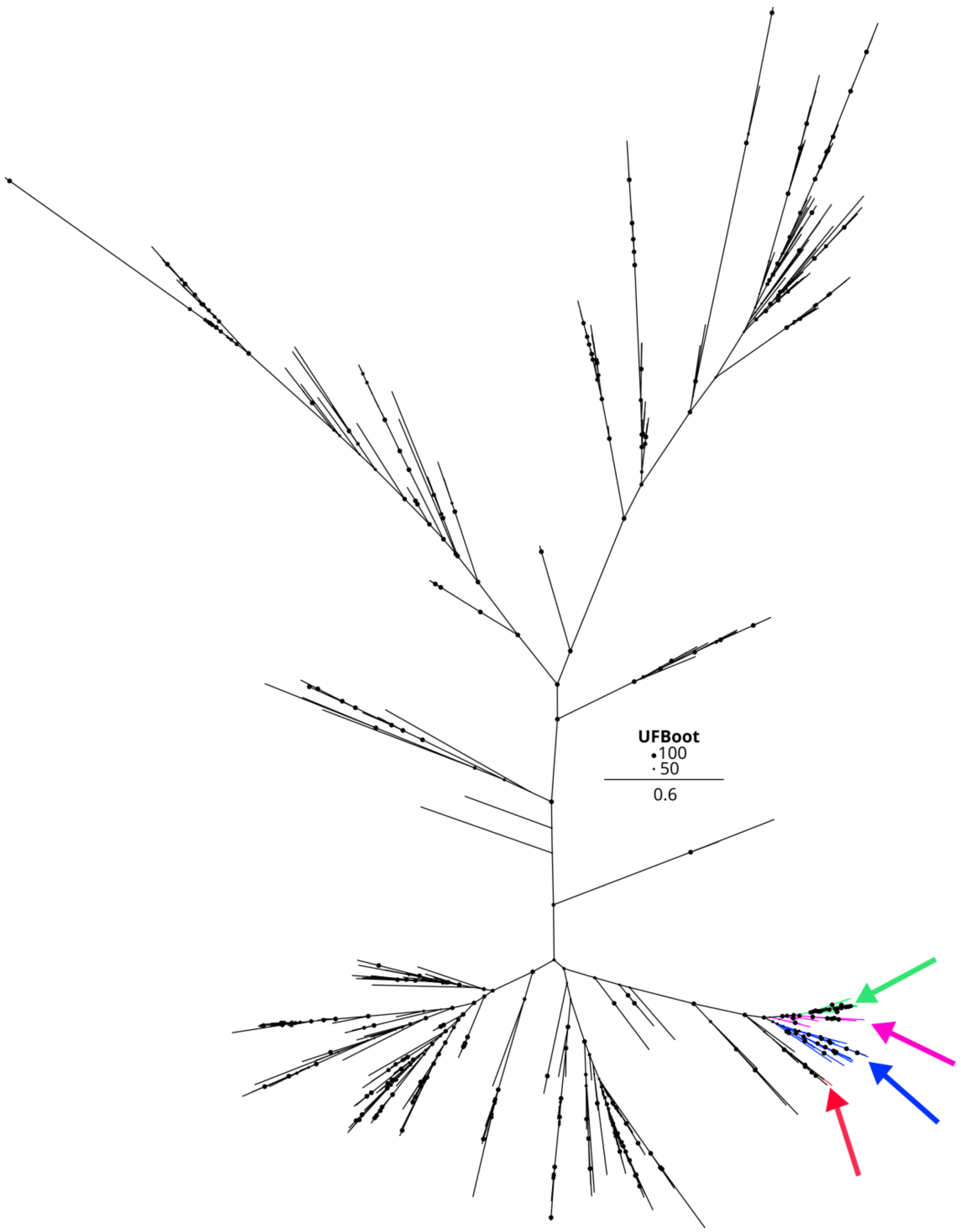

Figure S6: Maximum-likelihood phylogeny (unrooted) of cable bacteria DsrD and reference sequences. Phylogeny inferred using IQTree (Nguyen et al., 2015) According to the best-fit model (model Q.yeast+F+R6). Reference sequences are taken from a previously published study (Neukirchen and Sousa, 2021). Cable bacteria sequences (green) cluster with *Desulfobulbaceae* (pink) sequences. *Desulfocapsaceae* (blue) and *Desulfurivibrio alkaliphilus* (red) are more distantly related. DsrD sequences lack the oxidative type distinction.

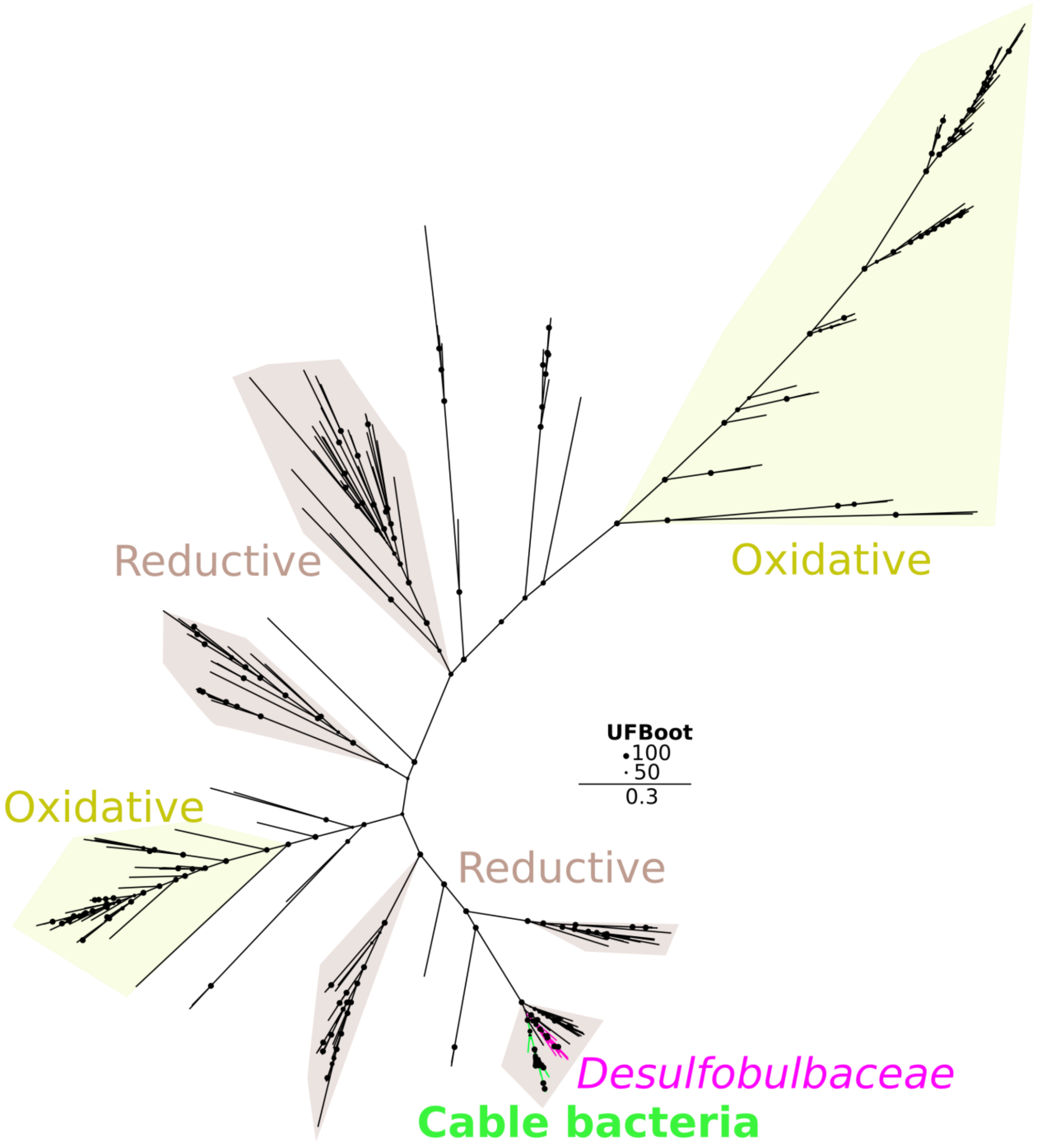

Figure S7: Maximum-likelihood phylogeny (unrooted) of cable bacteria AprA and reference sequences. Phylogeny inferred using IQTree (Nguyen et al., 2015) according to the best-fit model (model LG+F+I+R10). Reductive and oxidative branches are based on previously published data (Neukirchen and Sousa, 2021). Cable bacteria sequences cluster with *Desulfobulbaceae* sequences.

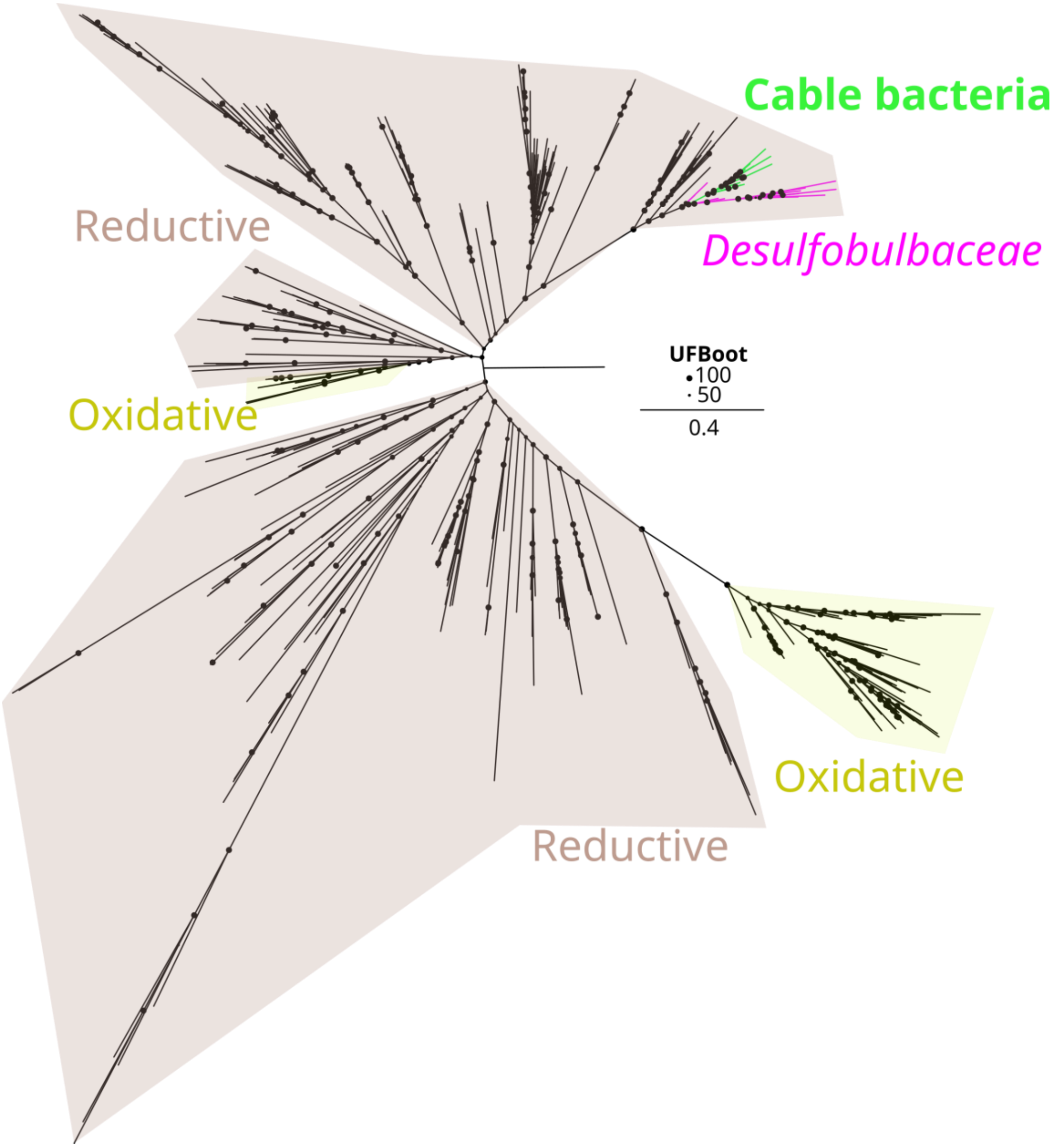

Figure S8: Maximum-likelihood phylogeny (unrooted) of cable bacteria AprB and reference sequences. Phylogeny inferred using IQTree (Nguyen et al., 2015) according to the best-fit model (model LG+F+I+R10). Reductive and oxidative branches are based on previously published data (Neukirchen and Sousa, 2021). Cable bacteria sequences cluster with *Desulfobulbaceae* sequences.

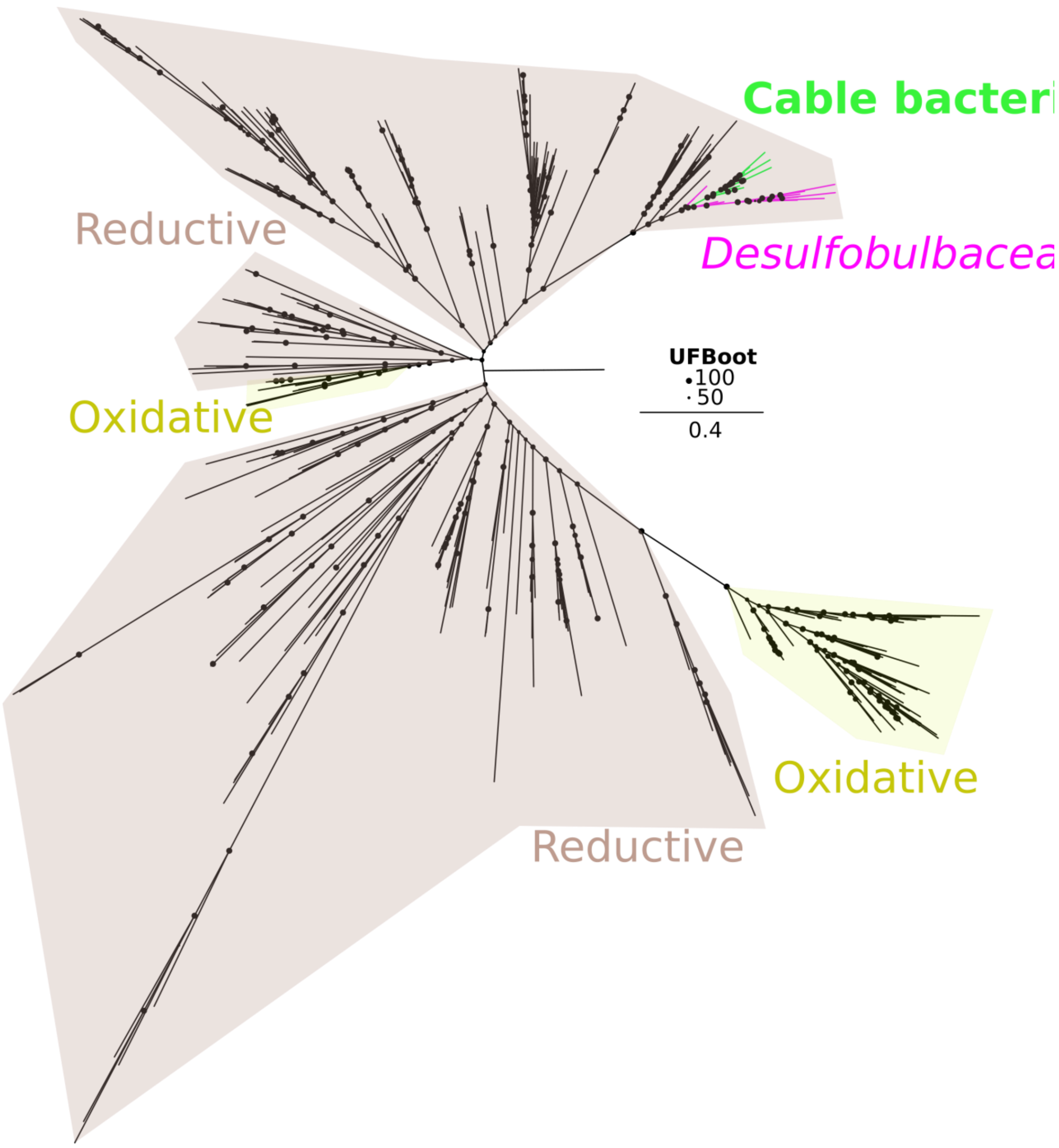

Figure S9: Maximum-likelihood phylogeny (unrooted) of cable bacteria QmoA and reference sequences. Phylogeny inferred using IQTree (Nguyen et al., 2015) according to the best-fit model (model LG+R10). Reductive and oxidative branches are based on previously published data (Neukirchen and Sousa., 2021). Cable bacteria sequences cluster with *Desulfobulbaceae* sequences.

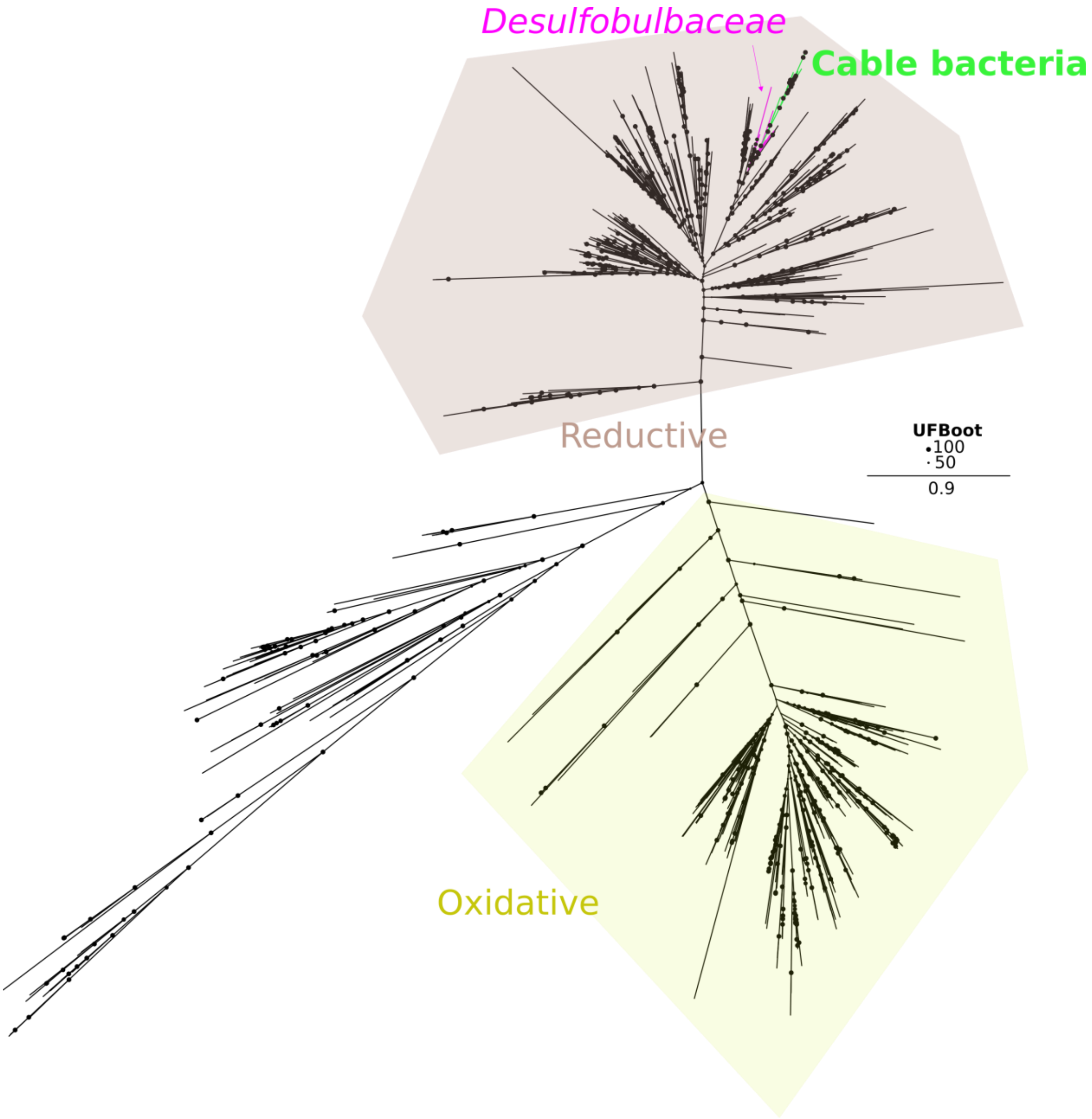

Figure S10: Maximum-likelihood phylogeny (unrooted) of cable bacteria DsrM and reference sequences. Phylogeny inferred using IQTree (Nguyen et al., 2015) according to the best-fit model (model LG+F+R10). Reductive and oxidative branches are based on previously published data (Neukirchen and Sousa., 2021). Cable bacteria sequences cluster with *Desulfobulbaceae* sequences.

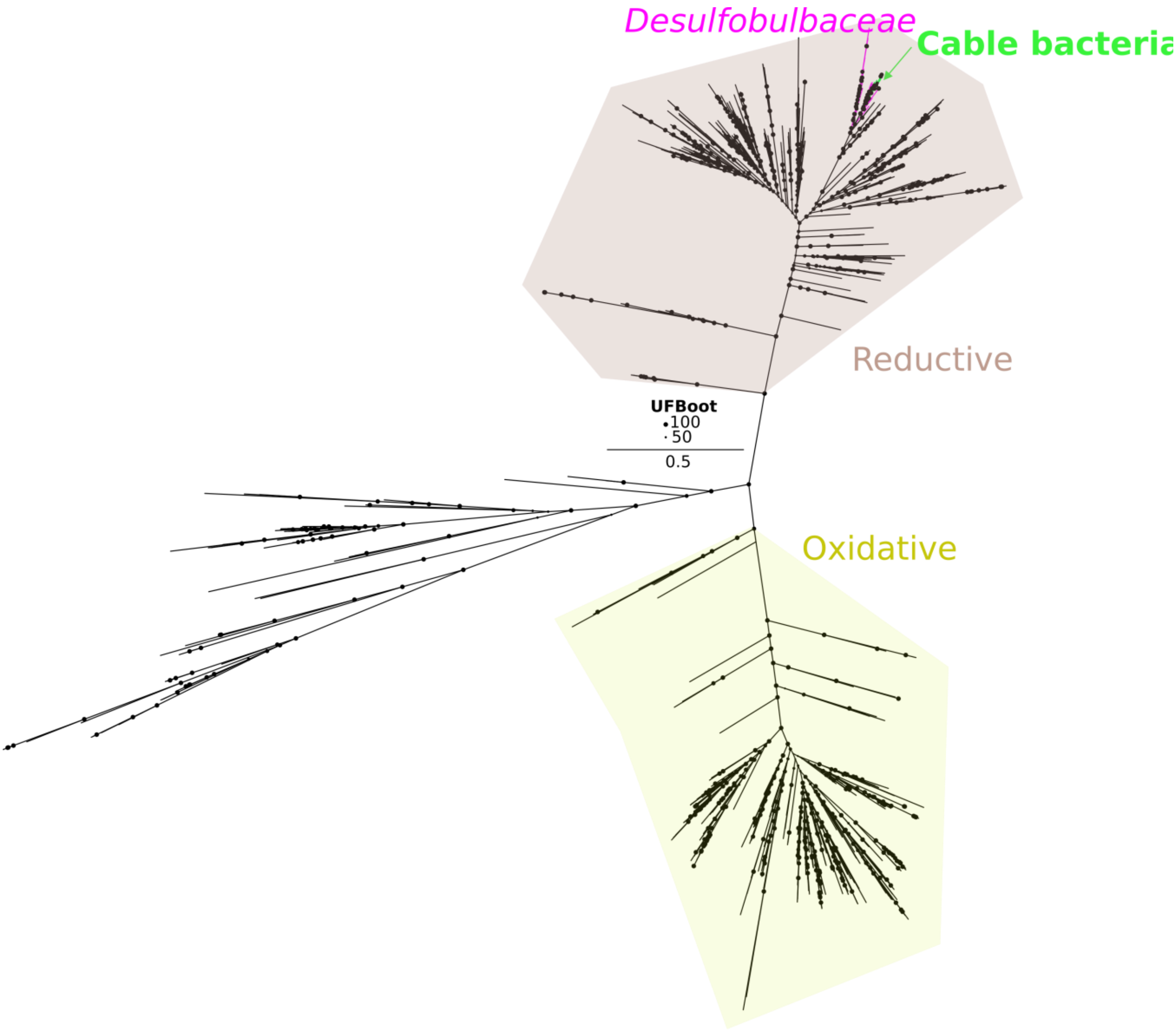

Figure S11: Maximum-likelihood phylogeny (unrooted) of cable bacteria DsrK and reference sequences. Phylogeny inferred using IQTree (Nguyen et al., 2015) according to the best-fit model (model LG+I+R10). Reductive and oxidative branches are based on previously published data (Neukirchen and Sousa., 2021). Cable bacteria sequences cluster with *Desulfobulbaceae* sequences.

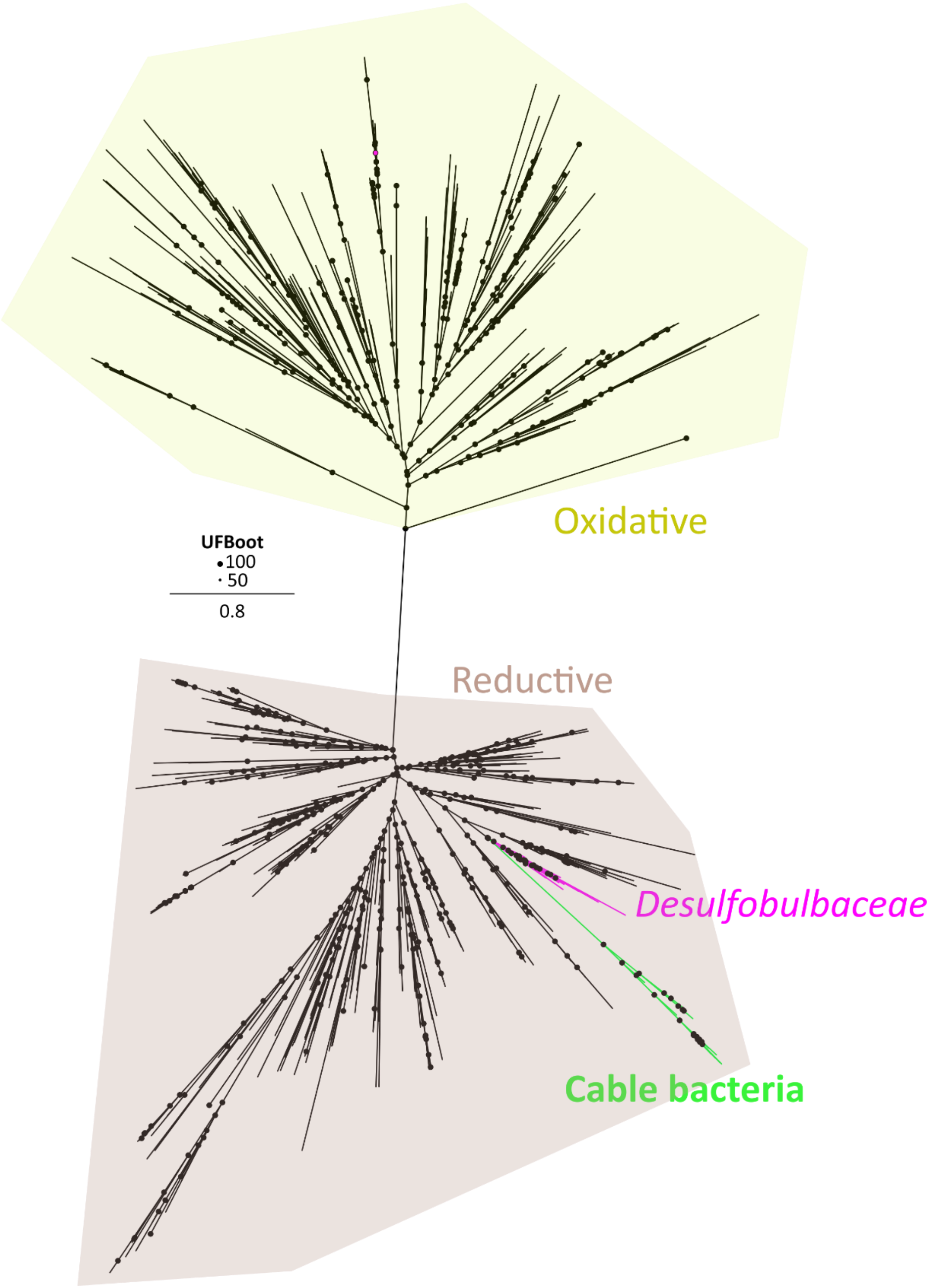

Figure S12: Maximum-likelihood phylogeny (unrooted) of cable bacteria DsrJ and reference sequences. Phylogeny inferred using IQTree (Nguyen et al., 2015) according to the best fit model (model LG+I+R10). Reductive and oxidative branches are based on previously published data (Neukirchen and Sousa., 2021). Cable bacteria sequences cluster with *Desulfobulbaceae* sequences, but form long branches.

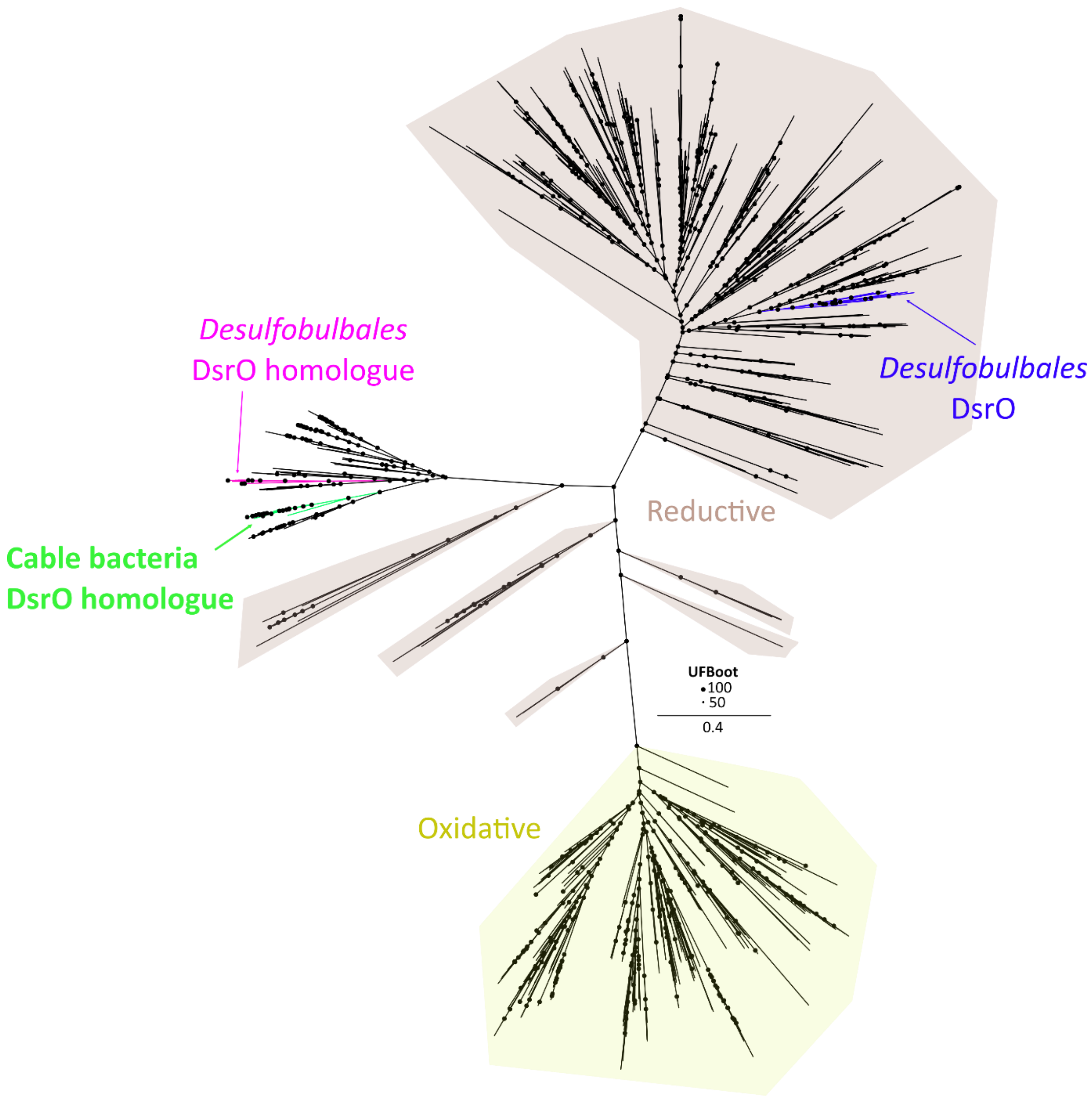

Figure S13: Maximum-likelihood phylogeny (unrooted) of cable bacteria DsrO and reference sequences. Phylogeny inferred using IQTree (Nguyen et al., 2015) according to the best fit model (model LG+F+R10). Reductive and oxidative branches are based on previously published data (Neukirchen and Sousa., 2021). Cable bacteria sequences cluster with sequences from *Gammaproteobacteria* and *Betaproteobacteria*. A putative second homolog of DsrO can be found in some *Desulfobulbales* (Figure 1A).

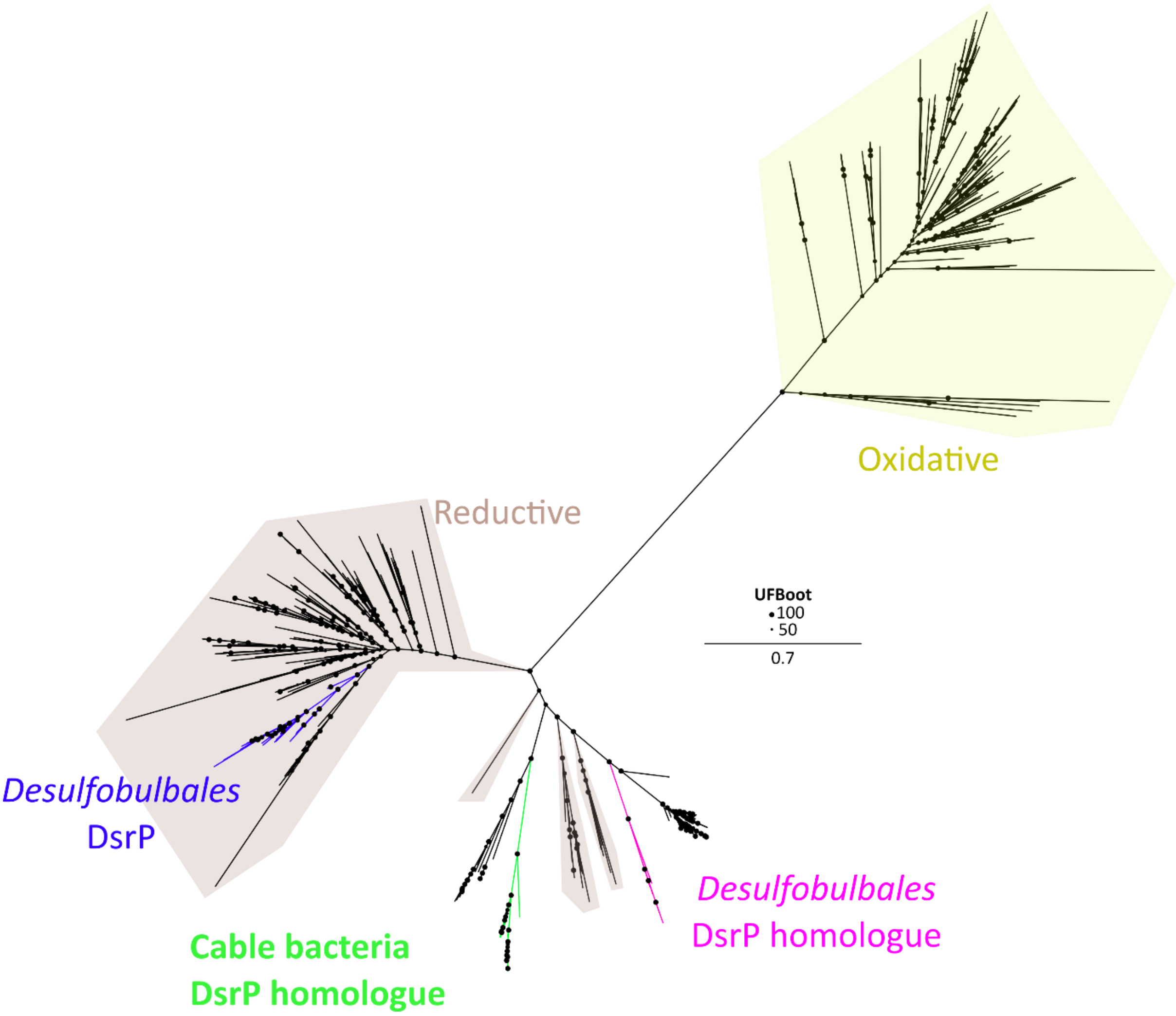

Figure S14: Maximum-likelihood phylogeny (unrooted) of cable bacteria DsrP and reference sequences. Phylogeny inferred using IQTree (Nguyen et al., 2015) according to the best-fit model (model LG+F+R10). Reductive and oxidative branches are based on previously published data (Neukirchen and Sousa., 2021). Cable bacteria sequences cluster with sequences from *Gammaproteobacteria* and *Betaproteobacteria*. A putative second homolog of DsrP can be found in some *Desulfobulbales* (Figure 1A).

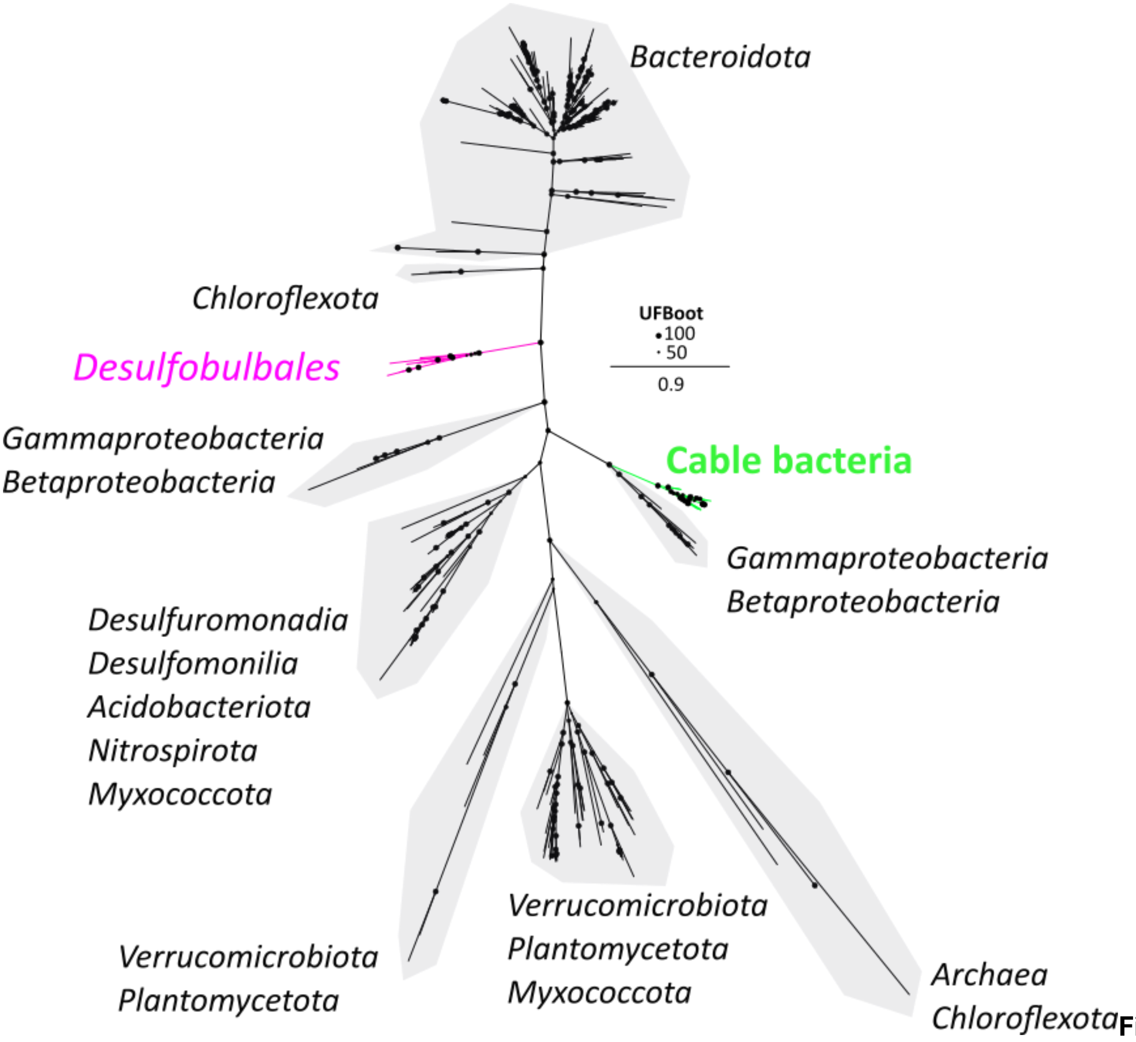

Figure S15: Maximum-likelihood phylogeny (unrooted) of cable bacteria tetraheam and reference sequences. Phylogeny inferred using IQTree (Nguyen et al., 2015) according to the best-fit model (model WAG+I+G4)). Cable bacteria sequences cluster with sequences from *Gammaproteobacteria* and *Betaproteobacteria*. A tetrahaem-containing protein can also be found in some Desulfobulbales (Figure 1A).

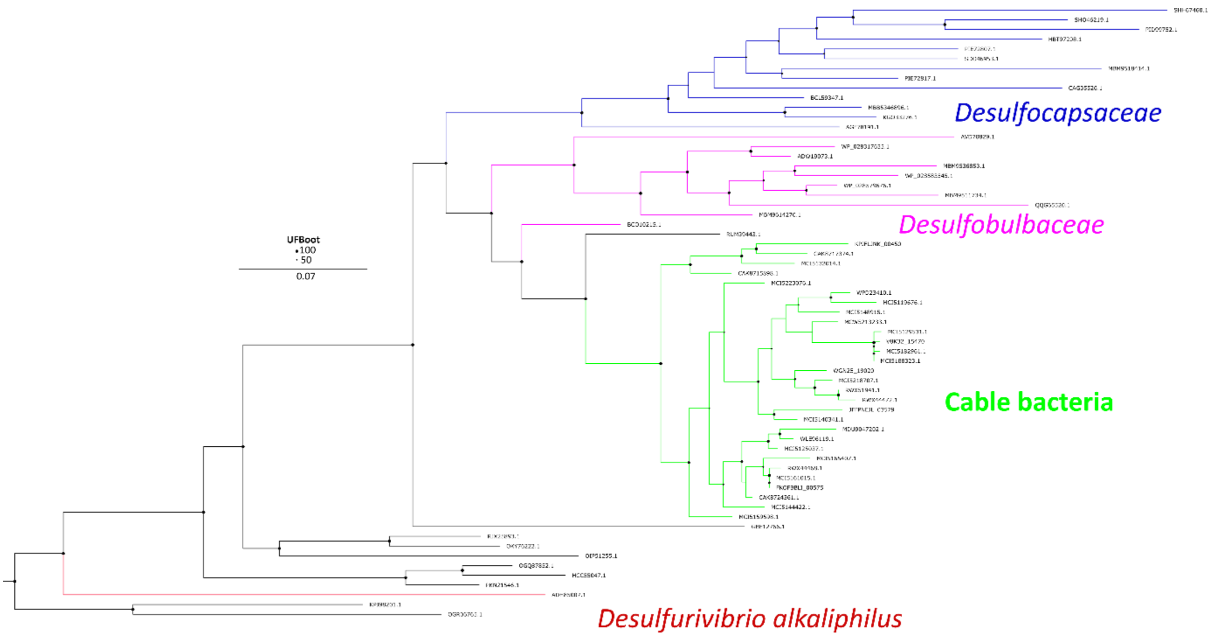

Figure S16: Zoom-in on maximum-likelihood phylogeny (unrooted) of cable bacteria DsrA and related sequences. Cable bacteria sequences cluster with *Desulfobulbaceae* sequences. *Desulfurivibrio alkaliphilus* DsrA is only distantly related. Protein accession numbers are indicated. Non-coloured branches are unclassified species.

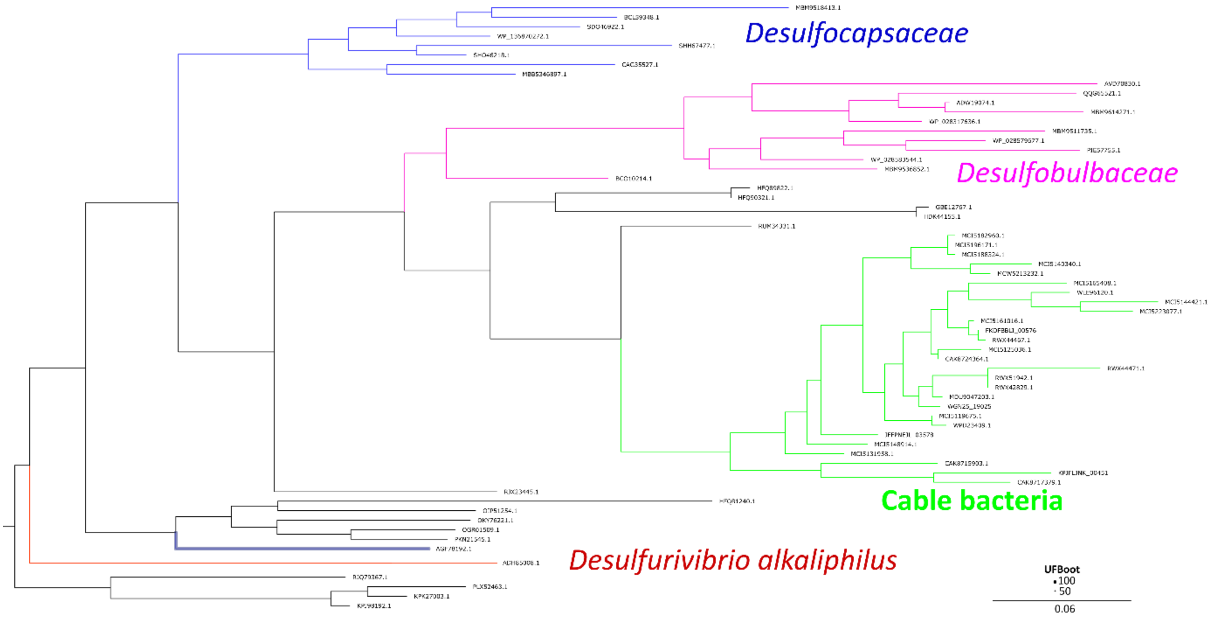

Figure S17: Zoom-in on maximum-likelihood phylogeny (unrooted) of cable bacteria DsrB and related sequences. Cable bacteria sequences cluster with *Desulfobulbaceae* sequences. *Desulfurivibrio alkaliphilus* DsrB is only distantly related. Protein accession numbers are indicated. Non-coloured branches are unclassified species.

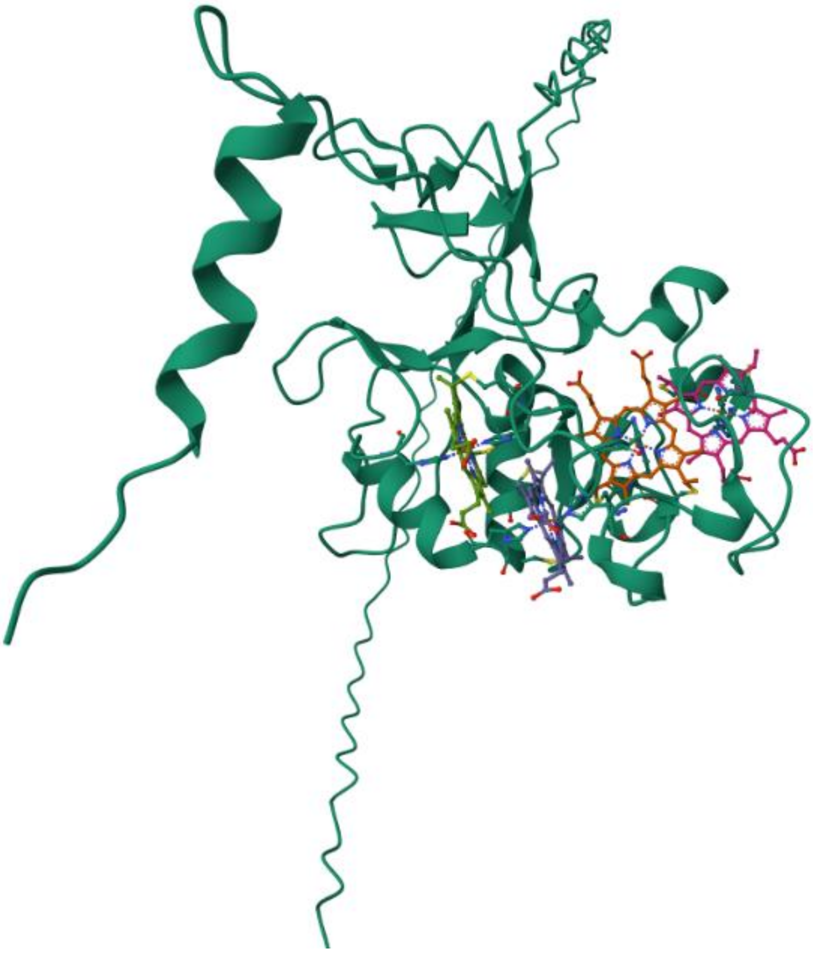

.

Figure S18: Structural prediction of periplasmic cytochrome *c* containing four haem-binding sites of *Ca*. Electrothrix scaldis GW3-3. Haem groups and histidines are indicated. All haem-groups are His-His coordinated. Structural prediction obtained using AlphaFold3 (Abramson et al., 2024).

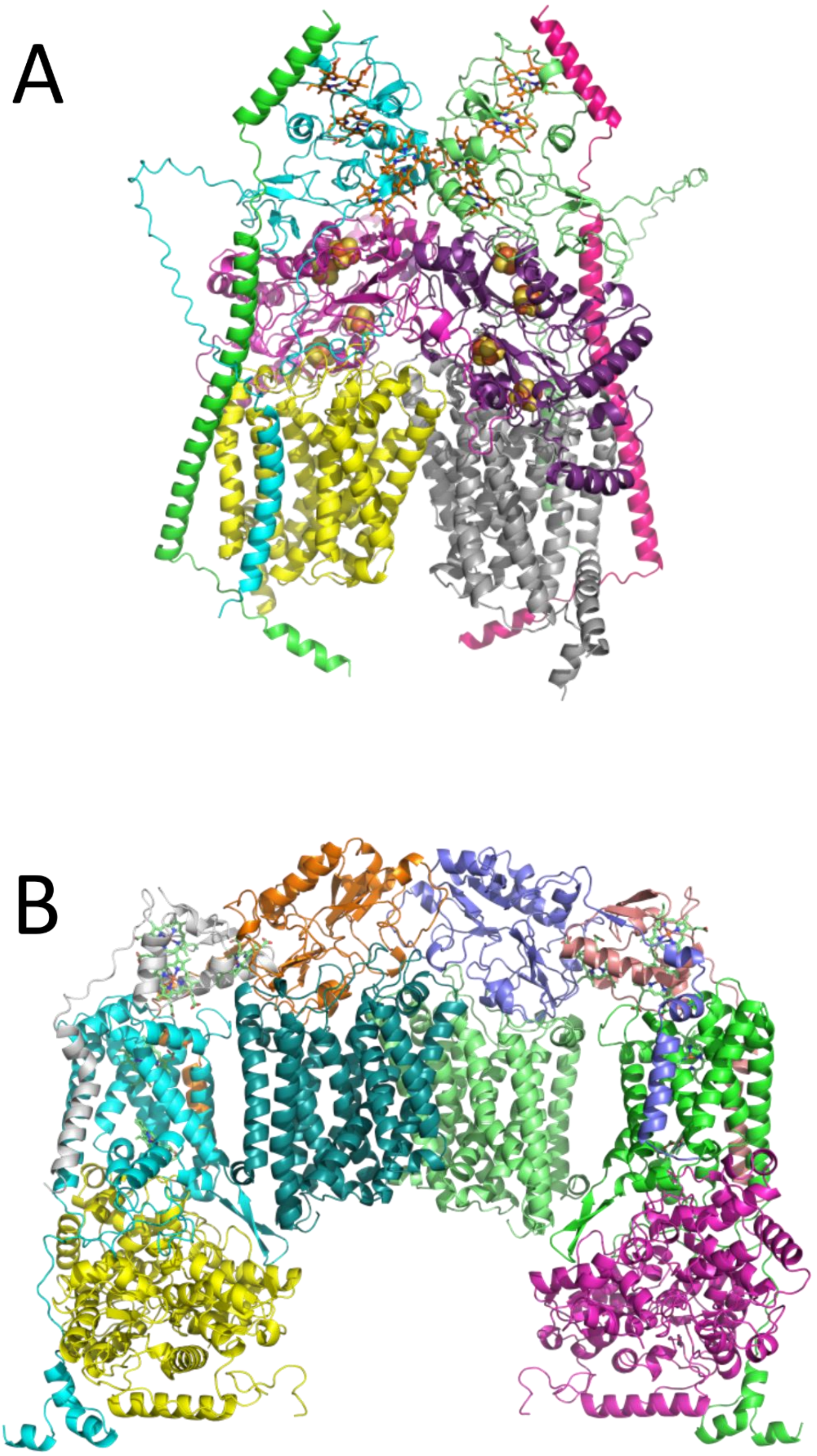

Figure S19: Structural prediction of the Dsr TMH_1-$DsrO_hP_h$-tetrahaem cluster of *Ca*. E. scaldis GW3-3 and the DsrMKJOP cluster of *Desulfovibrio vulgaris*. Structural predictions obtained using AlphaFold3 (Abramson et al., 2024).

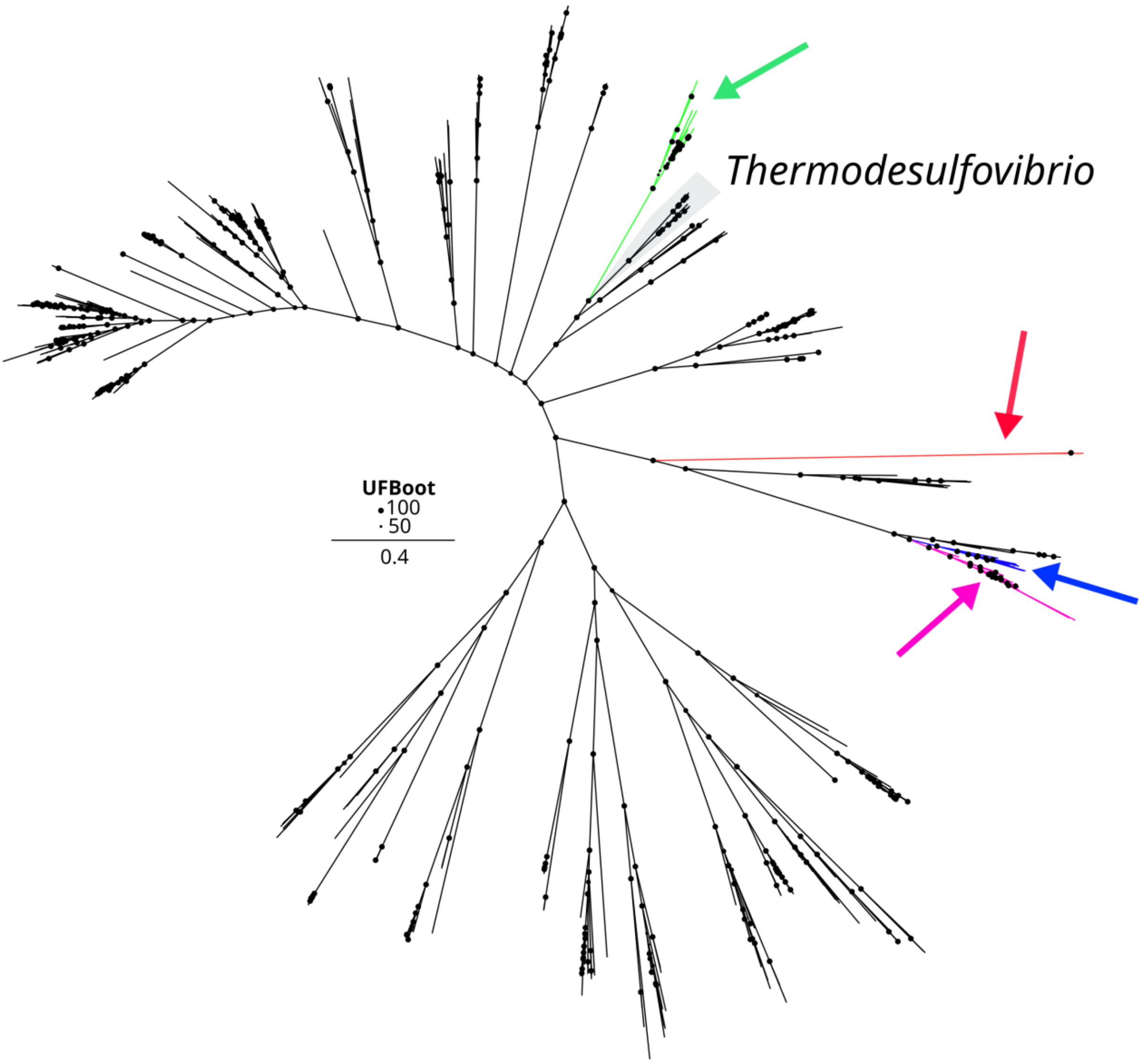

Figure S20: Maximum-likelihood phylogeny (unrooted) of cable bacteria PhsA/PsrA and RefSeq database hits. Phylogeny inferred using IQTree (Nguyen et al., 2015) according to the best-fit model (model LG+F+I+R8). Cable bacteria sequences (green) do not cluster with *Desulfobulbaceae (pink)*, *Desulfocapsaceae* (blue) nor *Desulfurivibrionaceae* (red) sequences, but rather within a clade of *Thermodesulfovibrio* (grey) genus sequences (*Nitrospirota* phylum).

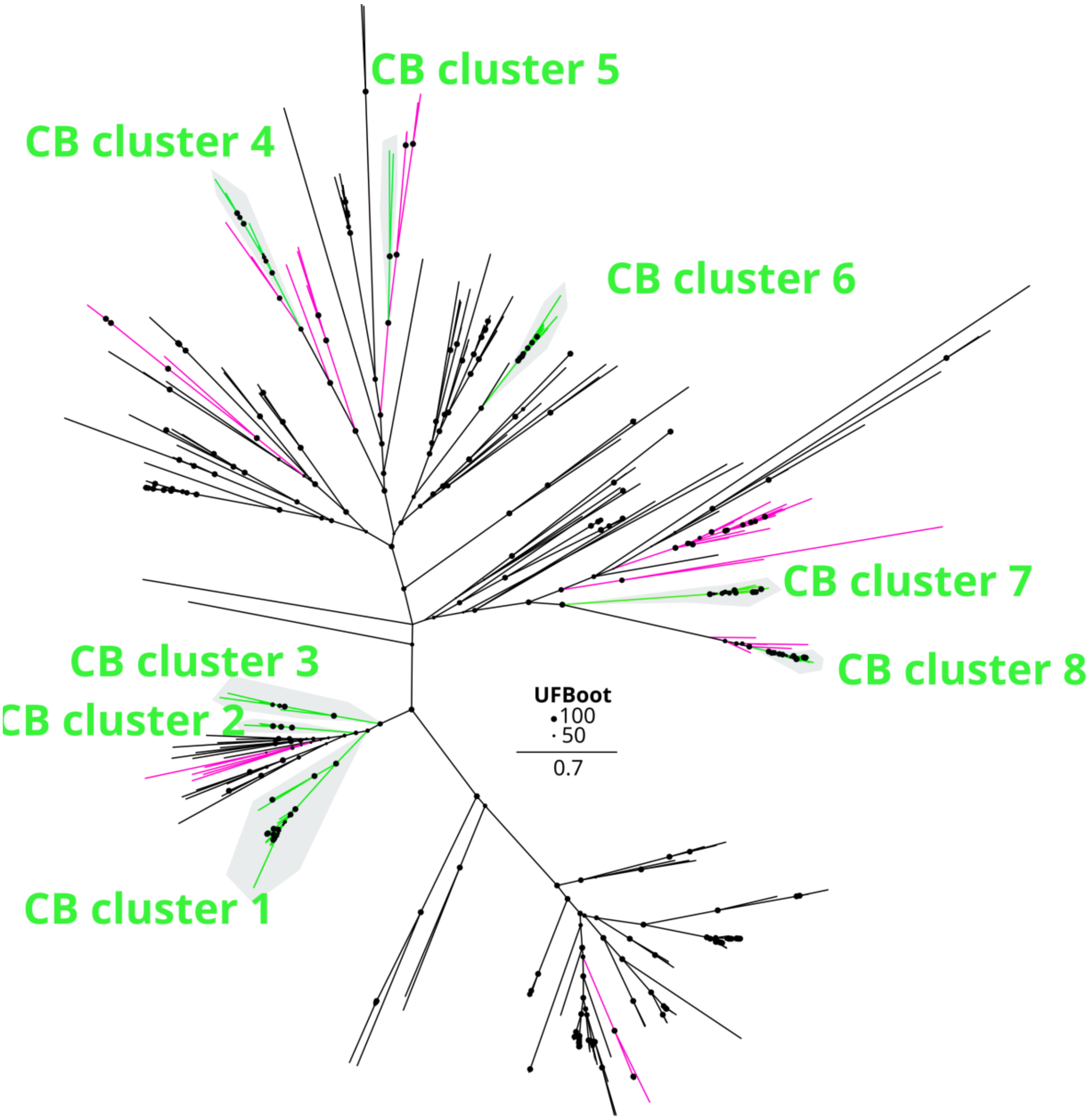

Figure S21: Maximum-likelihood phylogeny (unrooted) of cable bacteria rhodaneses and RefSeq database hits. Phylogeny inferred using IQTree (Nguyen et al., 2015) according to the best-fit model (model LG+R10). Cable bacteria clusters are indicated. Related *Desulfobulbaceae* sequences are indicated in pink.

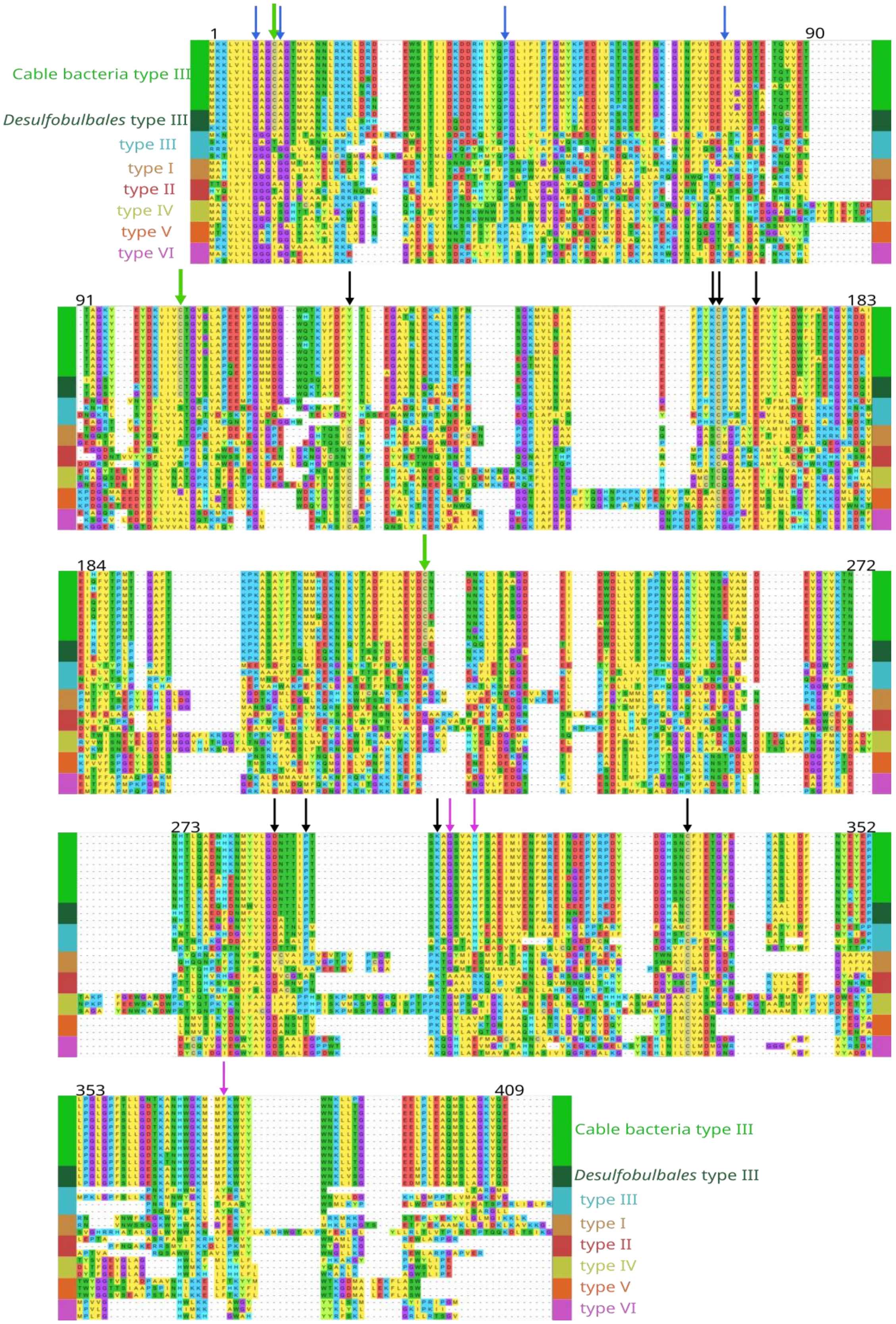

Figure S22: Multiple sequence alignment of sulphide:quinone reductases (SQRs) of cable bacteria sequences and references. Amino acid numbering (top) is based on *Ca*. Electrothrix gigas sp. GW3-3. Cable bacteria sequences and sequences from the *Desulfopila* and *Desulfolithobacter* genera possess three extra conserved cysteine residues (C11, C103, C226;

green arrows) compared to other SQR types (Marcia et al., 2010). Residues involved in sulphide oxidation (black arrows), FAD cofactor interaction (blue arrows) and quinone interaction (pink arrows) are indicated (Lencina et al., 2020, 2013; Marcia et al., 2010; Miklovics et al., 2022). Note that for the FAD interaction, the A12 and I78 residues (second and third blue arrow) are not identical to the characterized SQR of *Caldivirga maquilingensis* (where it is a G and A instead, respectively) (Lencina et al., 2020), but identical residues to cable bacteria SQRs are also found in other type III SQR sequences, indicating that FAD interaction is likely still functional. Cable bacteria sequences (from top to bottom) include *Ca*. E. gigas HY10-6 (WYD79468.1), *Ca*. E. sp. SY2, *Ca*. E. antwerpensis GW3-4 (WYD83751.1), *Ca*. E. communis RB (WLE96298.1), *Ca*. E. arhusiensis MCF (RWX44927.1), *Ca*. E. rattekaaiensis Rat1 (MDU9047309.1), *Ca*. E. sp. GW3-3 (WPD23765.1), *Ca*. En. halotolerans BRK-cMAG (CAK8712805.1), *Ca*. En. sp. SY1, *Ca*. En. aureum ENR-cMAG (CAK8719769.1). *Desulfobulbales* type III sequences include *Desulfolithobacter dissulfuricans* (BCO07960.1), *Desulfopila* sp. IMCC35008 (WP_163338798.1) and *Desulfopila inferna* (WP_205233531.1). Type III reference sequences include *Archaeoglobus fulgidus* (KUJ92657.1), *Chlorobaculum tepidum* (WP_010932704.1), *Caldivirga maquilingensis* (ABW01331.1), *Paramagnetospirillum magnetotacticum* (WP_009869729.1). Type I reference sequences include *Thiobacillus denitrificans* (WP_011312737.1), *Rhodobacter capsulatus* (WP_074553568.1), *Aphanothece halophytica* (AAF72963.1). Type II reference sequences *Pseudomonas auruginosa* (WP_033895535.1), *Staphylococcus aureus* (WP_001033019.1), *Ralstonia solanacearum* (WP_184849847.1). Type IV reference sequences *Chlorobaculum tepidum* (WP_010931811.1), *Allochromatium vinosum* (WP_012971338.1), *Sulfurimonas denitrificans* (WP_011372252.1). Type V reference sequences *Acidianus ambivalens* (CAD33806.), *Saccharolobus solfataricus* (AAK42426.1), *Sulfurisphaera tokodaii* (BAB67593.1). Type VI reference sequences include *Chlorobaculum tepidum* (WP_010932765.1), *Aquifex aeolicus* (WP_010880477.1), *Pelodictyon luteolum* (ABB23989.1).

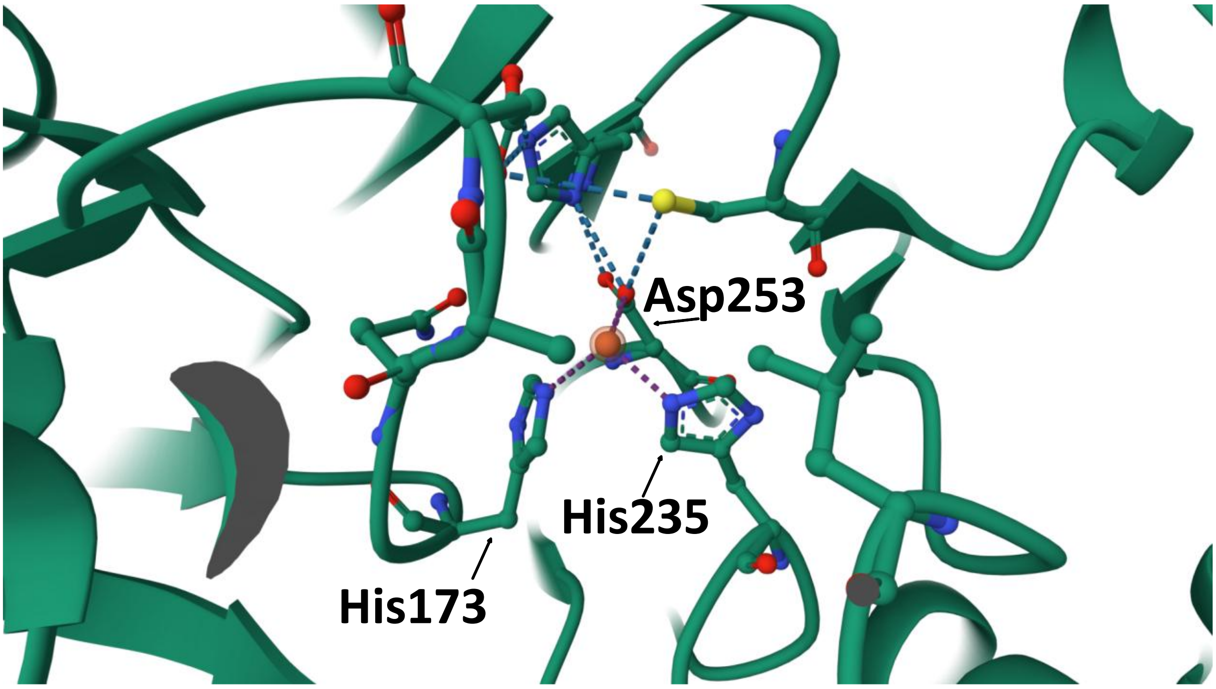

Figure S23: Zoom-in ofs tructural prediction of PDO of *Ca*. Electrothrix scaldis GW3-3. Iron ion (orange) is indicated along with the predicted binding residues. Structural prediction obtained using AlphaFold3 (Abramson et al., 2024).

Supplementary tables

Table S1: Overview of the genome dataset used in this study

Table S1 is available as a separate excel file

Table S2: locus tags of the genes identified in this study

Table S2 is available as a separate excel file

Table S3: *Gammaproteobacteria* and *Betaproteobacteria* sequences related to cable bacteria $DsrO_h$, $DsrP_h$, and tetrahaem sequences. For genome accessions see table S1.

Organism	*DsrO*	*DsrP*	*tetraheam*	*Dsr*	*S-Ox?*	*source*
Ferriphaselus amnicola	WP_197714099	WP_062625712	WP_062625713	Ox	no	Kato et al., 2014
Ca. Thiodiazotropha endolucinida	WP_069126785	WP_083220800	WP_069126787	Ox	yes	König et al., 2016
Ca. Thiodiazotropha sp. CDECU1	WP_316365646	WP_316365647	WP_316365648	Ox	yes	König et al., 2016
Ca. Thiodiazotropha *endoloripes*	WP_069006536	WP_069006535	WP_069006534	Ox	yes	König et al., 2016
Teredinibacter sp. KSP-S5-2	WP_316955586	WP_316955587	WP_316955588	-	?	
Sulfuritalea sp. UWPOB_SULF1	WP_294993986	WP_294993988	WP_294993990	Ox	yes	Kojima et al., 2011
Ideonella sp. A 288	WP_199873141	WP_088278991	WP_088278506	Ox	?	
Rhizobacter sp. AJA081-3	WP_209384477	WP_209384476	WP_209384475	Ox	?	
Piscinibacter sp.	WP_293417548	WP_293417545	WP_293417542	Ox	?	
Ca. Propionivibrio aalborgensis	WP_186410656	WP_186410655	WP_186410654	-	?	
Propionivibrio sp.	WP_294247222	WP_294247224	WP_294247226	-	?	
Sulfuricystis thermophila	WP_284155200	WP_172601743	WP_131110759	Ox	?	Kojima et al., 2022
Denitratisoma oestradiolicum	WP_197970470	WP_145770199	WP_145770198	-	?	
Sulfuritalea hydrogenivorans	WP_197539583	WP_052473601	WP_148312929	Ox	yes	Kojima et al., 2011
Sulfurisoma sediminicola	WP_121243245	WP_121243246	WP_124962403	Ox	yes	Kojima et al., 2014
Dechloromonas sp. HYN0024	WP_117608340	WP_117608341	WP_117608342	-	?	

References

Kato, S., Krepski, S., Chan, C., Itoh, T., Ohkuma, M., 2014. *Ferriphaselus amnicola* gen. nov., sp. nov., a neutrophilic, stalk-forming, iron-oxidizing bacterium isolated from an iron-rich groundwater seep. *Int J Syst Evol Microbiol* 64, 921–925. https://doi.org/10.1099/ijs.0.058487-0

Kojima, H., Fukui, M., 2014. *Sulfurisoma sediminicola* gen. nov., sp. nov., a facultative autotroph isolated from a freshwater lake. *Int J Syst Evol Microbiol* 64, 1587–1592. https://doi.org/10.1099/ijs.0.057281-0

Kojima, H., Fukui, M., 2011. *Sulfuritalea hydrogenivorans* gen. nov., sp. nov., a facultative autotroph isolated from a freshwater lake. *Int J Syst Evol Microbiol* 61, 1651–1655. https://doi.org/10.1099/ijs.0.024968-0

Kojima, H., Watanabe, M., Miyata, N., Fukui, M., 2022. *Sulfuricystis multivorans* gen. nov., sp. nov. and Sulfuricystis thermophila sp. nov., facultatively autotropic sulfur-oxidizing bacteria isolated from a hot spring, and emended description of the genus *Rugosibacter*. *Arch Microbiol* 204, 595. https://doi.org/10.1007/s00203-022-03186-0

König, S., Gros, O., Heiden, S.E., Hinzke, T., Thürmer, A., Poehlein, A., Meyer, S., Vatin, M., Mbéguié-A-Mbéguié, D., Tocny, J., Ponnudurai, R., Daniel, R., Becher, D., Schweder, T., Markert, S., 2016. Nitrogen fixation in a chemoautotrophic lucinid symbiosis. *Nat Microbiol* 2, 16193. https://doi.org/10.1038/nmicrobiol.2016.193

Lencina, A.M., Ding, Z., Schurig-Briccio, L.A., Gennis, R.B., 2013. Characterization of the Type III sulfide:quinone oxidoreductase from Caldivirga maquilingensis and its membrane binding. *Biochimica et Biophysica Acta (BBA) - Bioenergetics* 1827, 266–275. https://doi.org/10.1016/j.bbabio.2012.10.010

Marcia, M., Ermler, U., Peng, G., Michel, H., 2010. A new structure-based classification of sulfide:quinone oxidoreductases. Proteins: Structure, Function, and Bioinformatics 78, 1073–1083. https://doi.org/10.1002/prot.22665

Marcia, M., Ermler, U., Peng, G., Michel, H., 2009. The structure of *Aquifex aeolicus* sulfide:quinone oxidoreductase, a basis to understand sulfide detoxification and respiration. Proceedings of the National Academy of Sciences 106, 9625–9630. https://doi.org/10.1073/pnas.0904165106

Miklovics, N., Duzs, Á., Balogh, F., Paragi, G., Rákhely, G., Tóth, A., 2022. Quinone binding site in a type VI sulfide:quinone oxidoreductase. Appl Microbiol Biotechnol 106, 7505–7517. https://doi.org/10.1007/s00253-022-12202-8
